# USP28 promotes PARP inhibitor resistance by enhancing SOX9-mediated DNA damage repair in ovarian cancer

**DOI:** 10.1038/s41419-025-07647-4

**Published:** 2025-04-16

**Authors:** Fang Han, Gonghua Qi, Rongrong Li, Jiali Peng, Shi Yan, Cunzhong Yuan, Beihua Kong, Hanlin Ma

**Affiliations:** 1 Department of Obstetrics and Gynecology, Qilu Hospital of Shandong University, Jinan, China; 2 Gynecologic Oncology Key Laboratory of Shandong Province, Qilu Hospital of Shandong University, Jinan, China; 3 Department of Ophthalmology, Qilu Hospital of Shandong University, Jinan, China

**Keywords:** Cancer therapeutic resistance, Ovarian cancer

## Abstract

PARP inhibitor (PARPi) resistance presents a significant challenge in ovarian cancer treatment, necessitating the development of effective therapeutic strategies to overcome this resistance and improve patient outcomes. Our study demonstrated that elevated expression of SRY-box 9 (SOX9) contributes to olaparib resistance in ovarian cancer. Mechanistically, the deubiquitinating enzyme USP28 was identified as a novel interacting partner of SOX9. USP28 inhibited the ubiquitination and subsequent degradation of SOX9, which is mediated by the E3 ubiquitin ligase FBXW7 during olaparib treatment. ChIP-Seq analysis revealed that SOX9 binds to the promoters of key DNA damage repair (DDR) genes (*SMARCA4*, *UIMC1*, and *SLX4*), thereby regulating DDR processes in ovarian cancer. Additionally, USP28 promoted olaparib resistance by stabilizing SOX9 protein and enhancing DNA damage repair. Furthermore, the USP28 specific inhibitor AZ1 reduced SOX9 protein stability and increased the sensitivity of ovarian cancer cells to olaparib. In conclusion, targeted inhibition of USP28 promoted ubiquitination-mediated degradation of SOX9, thereby impairing DNA damage repair capabilities and sensitizing ovarian cancer cells to PARPi. These findings elucidate the underlying mechanisms of PARPi resistance in ovarian cancer and suggest the potential efficacy of combining USP28 inhibitors with PARPi to overcome this resistance.

## Introduction

Ovarian cancer stands as the most lethal gynecological malignancy worldwide. According to statistics from the American Cancer Society, an estimated 12740 deaths are projected to occur in the United States in 2024 [1]. Early-stage ovarian cancer typically presents without obvious symptoms and lacks reliable screening methods. Consequently, approximately 70% of patients are diagnosed at advanced stages (III-IV), with a 5-year survival rate of less than 30% [2]. Epithelial ovarian cancer (EOC) accounts for about 90% of ovarian cancer cases, with high-grade serous ovarian carcinoma (HGSOC) being the most common subtype within this category, responsible for about 70% of all ovarian cancer-related deaths [3, 4]. Platinum-based chemotherapy is the standard treatment for HGSOC following surgical cytoreduction. However, only about 20% of advanced-stage patients exhibit a favorable response and achieve long-term survival, while the remaining 80% experience disease recurrence within two years and have limited treatment options [5].

In 2014, olaparib became the first poly (ADP-ribose) polymerase inhibitor (PARPi) to be approved by the FDA as a maintenance therapy for recurrent EOC carrying BRCA mutations [6]. Subsequently, two other PARPis, rucaparib and niraparib, received regulatory approval for application across various histologies characterized by homologous recombination deficiency (HRD) or BRCA mutations [7]. PARPi has demonstrated unprecedented therapeutic efficacy, significantly prolonging progression-free survival (PFS) and overall survival (OS) in ovarian cancer patients. However, a considerable portion of patients are inherently resistant to PARPi, and even those initially responsive often face disease progression due to the emergence of acquired drug resistance [8, 9]. Therefore, it is urgent to elucidate the molecular mechanisms driving ovarian cancer resistance to PARPi and to develop new combination therapy strategies.

Transcription factor sex determining region Y (SRY)-box 9 (SOX9), a member of the SOX (SRY-type HMG box) family, was initially discovered to play crucial roles in embryonic development and cellular stemness [10]. Recently, SOX9 has emerged as a promising therapeutic target, as evidenced by its identification through CRISPR-Cas9 screens of human cancers [11]. Accumulating studies reveal that SOX9 is highly expressed in various cancers, and its upregulation correlates with enhanced cancer cell proliferation, invasion, and metastasis [12]. In addition, elevated SOX9 expression favors the development of therapy resistance in various cancers, consequently leading to unfavorable prognostic outcomes [12, 13]. SOX9, acting as a downstream factor of SOX2, contributes to tamoxifen resistance by regulating ALDH1A3 expression and modulating Wnt signaling in breast cancer [14]. Another study has reported that SOX9 is involved in ATP-driven invasion and chemoresistance by targeting CEACAM5/6, ABCB1, and ABCG2 in breast cancer [15]. SOX9 is notably upregulated in ovarian cancer tissues, and its high expression is indicative of poor prognosis, lymph node metastasis, and chemotherapy resistance [16]. Moreover, the overexpression of SOX9 has been shown to stimulate metastasis and proliferation of ovarian cancer cells [17, 18]. The aberrant expression of SOX9 has also been associated with cisplatin (CDDP) resistance in ovarian cancer cells [19]. However, the function of SOX9 in the PARPi resistance of ovarian cancer remains unclear.

In the current study, we report for the first time that SOX9 is positively correlated with PARPi resistance in ovarian cancer. Further mechanistic studies indicate that targeted inhibition of USP28 using a specific inhibitor leads to ubiquitination-mediated degradation of SOX9, consequently inhibiting DNA damage repair capabilities, and ultimately enhancing the sensitivity of ovarian cancer to PARPi. Our investigation elucidates the mechanisms of PARPi resistance in ovarian cancer, thereby providing potential strategies for developing new combination therapies to overcome this resistance.

## Materials and methods

### Cell lines and cell culture

The SKOV3 and UWB1.289 ovarian cancer cell lines were acquired from the American Type Culture Collection (ATCC, Manassas, VA, USA). The HEK293T cell line was sourced from the Cell Bank of the Chinese Academy of Sciences (Shanghai, China). PARPi-resistant SKOV3 (SKOV3/Ola) cell line was generated by treating parental SKOV3 cells with increasing concentrations of olaparib in our lab, as described previously [20]. UWB1.289 cells were cultured in RPMI 1640 medium (Gibco, Grand Island, NY, USA). SKOV3 and SKOV3/Ola cell lines were maintained in McCoy’s 5A medium (Gibco). HEK293T cells were maintained in high-glucose Dulbecco’s Modified Eagle Medium (DMEM, Gibco). The culture media were supplemented with 10% fetal bovine serum (FBS, Gibco) and 1% penicillin/streptomycin (15140-122, Gibco). All cell lines were routinely screened for mycoplasma contamination and authenticated by short tandem repeat (STR) profiling. All cells were grown in a humidified incubator at 37 °C with 5% CO_2_.

### Antibodies and reagents

Antibodies against GST (10000-0-AP), poly (ADP-ribose) polymerase 1 (PARP1, 13371-1-AP), His (66005-1-Ig), Flag (20543-1-AP), MYC (16286-1-AP), HA (81290-1-RR), SMARCA4 (21634-1-AP), USP25 (12199-1-AP), and USP28 (17707-1-AP) were procured from Proteintech (Wuhan, China). Antibodies for β-actin (A5441) and SOX9 (AB5535) were obtained from Sigma-Aldrich (St. Louis, MO, USA). Ubiquitin (sc-8017) antibody was sourced from Santa Cruz Biotechnology (Dallas, TX, USA). PAXIP1 (A17431) antibody was purchased from ABclonal (Wuhan, China). The mouse IgG (A7028) and rabbit IgG (A7016) were purchased from Beyotime (Shanghai, China). Antibodies against FBXW7 (ab192328), UIMC1 (ab124763), Ki-67 (ab245113), γH2AX (ab81299), H2AX (ab124781), RAD51 (ab133534), and SLX4 (ab169114) were obtained from Abcam (Cambridge, UK). Goat anti-rabbit IgG Alexa Fluor-488 (A-11008) for immunofluorescence staining was obtained from Invitrogen (Waltham, MA, USA). The olaparib (AZD2281), AZ1 (S8904), Cycloheximide (CHX, S7418), and MG132 (S2619) were purchased from Selleck Chemicals (Houston, TX, USA).

### Western blot

The ovarian cancer cells were rinsed with PBS and lysed in RIPA buffer (P0013B, Beyotime) containing 1 × Protease Inhibitor Cocktail (P1005, Beyotime). The cell supernatants were collected after centrifugation at 12000 rpm for 5 min. Protein concentration was quantified using the BCA Protein Assay Kit (P0012, Beyotime). Protein lysates (20–40 µg) were resolved on 8–15% polyacrylamide gels and transferred onto PVDF membranes (Merck Millipore, Burlington, MA, USA). Membranes were then sequentially incubated with 5% fat-free milk, 1:1000 of the indicated primary antibodies, and 1:5000 of the HRP-conjugated secondary antibodies. The protein bands were visualized using an enhanced ECL detection kit (ORT2655, PerkinElmer, Waltham, MA, USA) and the GE Amersham Imager 600 (GE, Chicago, IL, USA). The grayscale intensities of the bands were assessed using ImageJ 1.52a.

### Co-immunoprecipitation (Co-IP)

Cells were washed twice with PBS and lysed using Western and IP Lysis Buffer (P0013B, Beyotime) with 1 × Protease Inhibitor Cocktail (P1005, Beyotime). The cellular supernatants were obtained following centrifugation at 12000 rpm for 5 min. Subsequently, 800 μg of the cellular extract was co-incubated with 5 µL of primary antibodies or normal rabbit/mouse IgG overnight, followed by incubation with protein A/G magnetic beads (B23202, Selleck) for 2 h on a rotary shaker at 4 °C. After three washes with Western and IP Lysis Buffer, the bound immune complexes were boiled in 2 × SDS loading buffer and subjected to western blot analysis. The anti-HA (KTSM1335, AlpaLifeBio, Shenzhen, China) Nanobody Magarose Beads, anti-Flag (KTSM1338, AlpaLifeBio) Beads, and HRP-conjugated Mouse anti-Rabbit IgG Light Chain (AS061, ABclonal, Wuhan, China) were used to avoid interference from denatured IgG.

### Mass spectrometry (MS) assay

UWB1.289 cells stably transfected with pCMV (Flag-tag) or pCMV SOX9 (Flag-SOX9) were lysed in Western and IP Lysis Buffer. After centrifugation, the cleared lysates were incubated with anti-Flag Nanobody Magarose Beads overnight. The beads were then washed three times with lysis buffer and denatured using SDS loading buffer. Then, the immunoprecipitated (IPed) proteins were separated on SDS-PAGE gels and silver-stained. Gel slices showing the most significant differences in band intensity between the two groups were excised and sent for analysis. The LC-MS/MS analysis was performed by APTBIO company (Shanghai, China). The list of interaction proteins of SOX9 was provided in Supplementary Table S1.

### Chromatin immunoprecipitation (ChIP) assay

The ChIP-seq assay was conducted and analyzed by Xiuyue Technologies (Jinan, China). The ChIP-qPCR assay was performed using the EZ-ChIP Chromatin immunoprecipitation Kit according to the manufacturer’s instructions (17–371, Merck KGaA, Darmstadt, Germany). In brief, UWB1.289 cells were cross-linked with 37% formaldehyde, washed with cold 1×PBS, and collected into 1.5 mL EP tubes. The cells were then lysed in lysis buffer and DNA was sheared into ~200–1000 bp fragments by sonication. 10 µL supernatant was retained as input control. The DNA-protein complexes were then immunoprecipitated using SOX9 antibody (AB5535, Sigma-Aldrich) or negative control IgG. After the cross-links were reversed, the associated DNA fragments were eluted, and analyzed by qRT-PCR. The primer sequences used for ChIP-qPCR are listed in Supplementary Table S2 (Supporting Information). The ChIP-seq datasets have been deposited at the Gene Expression Omnibus (GEO) with the accession number GSE271296.

### Plasmid construction

The lentivirus overexpression plasmids (pCMV SOX9, pCMV USP28) were created by inserting the open reading frames (ORFs) of human SOX9 and USP28 into the pCMV vector (PS100069, OriGene, Rockville, MD, USA). The lentivirus knockdown plasmids (SOX9 shRNA-1, SOX9 shRNA-2, USP28 shRNA-1, USP28 shRNA-2, USP25 shRNA) were generated by cloning the shRNA duplex sequences into the pLKO.1-puro vector (8453, Addgene, Watertown, MA, USA). The shRNA sequences targeting SOX9, USP28, and USP25 are detailed in Supplementary Table S3 (Supporting Information). HA-USP28, Flag-SOX9, and Myc-FBXW7 were produced by inserting the ORFs into pcDNA3.1 (Addgene) vectors with HA-tag, Flag-tag, or Myc-tag, respectively. DNA sequencing was used to validate the accuracy of constructs.

### Transfection

Plasmid transfection was performed using the Lipofectamine 2000 reagent (11668-019, Invitrogen) following the manufacturer’s protocols. Lentivirus particles were generated in HEK293T cells by packaging lentivectors with psPAX2 and pMD2.G. To establish stable cell lines, cells were subjected to lentiviral infection for 24 h, followed by selection in media containing 2 μg/mL puromycin (P8833, Sigma-Aldrich) for two weeks. The efficiency of gene knockdown and overexpression was verified using western blot analysis.

### Annexin V APC/7-AAD assay for apoptosis

The apoptosis assay was conducted using Annexin V APC Apoptosis Detection Kit (62700-80, BioGems, Westlake Village, CA, USA). Briefly, cells were trypsinized, washed with PBS, and resuspended in 1×Annexin V Binding Buffer. Then single-cell suspensions (1 × 10^6^) were stained with Annexin V APC (5 µL) for 5 min and 7-AAD (5 µL) for 15 min on ice in the dark. The stained cells were acquired using a BD FACSCalibur flow cytometer and the data were analyzed using FlowJo v10.6.2 software.

### Colony formation assay

Cells (600 cells per well) were seeded in 6-well plates as single-cell suspensions. After the indicated treatment, the colonies were fixed with methanol for 15 min and stained with crystal violet for 15 min. The colonies were then photographed, and ImageJ 1.52a software was used to count the number of colonies (>50 cells).

### MTT assay

Cells (3000 cells per well) were seeded into 96-well plates. After treatment, 20 µL of MTT solution was added to each well. After incubating for 4 h at 37 °C, the medium was discarded and 200 μl of DMSO was added. The plate was then gently vortexed in the dark for 10 min. The absorbance at 490 nm was measured using a Thermo Scientific Microplate Reader (Waltham, MA, USA).

### Immunohistochemistry (IHC) staining

Tumor tissues were isolated and immediately fixed in formalin solution overnight. Subsequently, the samples were paraffin-embedded, sectioned into 4 μm slices, deparaffinized, and rehydrated using graded ethanol, followed by antigen retrieval with EDTA solution. Then, the slides were subjected to standard IHC staining using a Universal Two Step Kit (PV9000, ZSGB-BIO, Beijing, China). Visualization was performed using DAB (ZSGB-BIO), followed by counterstaining with hematoxylin. Stained sections were observed and captured using an Olympus microscope (Tokyo, Japan). Expression levels in each sample were evaluated by two pathologists based on the intensity and extent of staining. The intensity of staining was scored as 0 (negative), 1 (weak), 2 (moderate), or 3 (strong). The extent of staining was determined based on the percentage of positive tumor cells: 1 (0–25%), 2 (26–50%), 3 (51–75%), and 4 (76–100%). The final score for each sample was the average of the scores from the two duplicates.

### Immunofluorescence (IF) staining

Cells were seeded into 15 mm glass-bottom cell culture dish (NEST Biotech, Wuxi, China), After olaparib treatment, the cells were washed with PBS, fixed with 4% paraformaldehyde for 15 min, permeabilized with 0.2% Triton X-100 solution for 5 min, and blocked with normal goat serum for 30 min. Subsequently, the cells were incubated with RAD51 (1:100) or γH2AX (1:100) primary antibodies overnight, and Goat anti-rabbit IgG Alexa Fluor-488 (A-11008) secondary antibody (1:200) for 1 h in the dark. Cells were then counterstained with DAPI to highlight nuclei. Images were captured using a Sunny IRX-60 confocal microscope (Sunny Technology, Beijing, China).

### qRT-PCR

Total RNA samples were extracted with TRIzol reagent (15596018, Invitrogen) following the manufacturer’s instructions. Subsequently, 1 μg of total RNAs was reverse transcribed into cDNA using the PrimeScript RT reagent Kit (RR037A, TaKaRa, Kyoto, Japan). Real-time PCR was performed with the SYBR Premix Ex Taq (RR420A, TakaRa) system on the 7900HT Fast Real-Time PCR machine (Applied Biosystems, Waltham, MA, USA). The mRNA levels of specific genes were calculated with the comparative Ct method (2^-ΔΔCt^), with β-actin serving as a housekeeping gene. The primer sequences used for qRT-PCR are listed in Supplementary Table S4 (Supporting Information).

### GST pull-down assay

The coding sequences of SOX9 and USP28 were cloned into the pGEX-4T-1 and pET-22b(+) vectors to generate the GST-SOX9 and His-USP28 constructs, respectively. The GST-SOX9 and His-USP28 plasmids were then transfected into *Escherichia coli* BL21. Protein expression was induced with 1 mM IPTG. The GST-SOX9 protein was purified using the GST-tag Protein Purification Kit (P2262, Beyotime), and His-USP28 was purified with His-Tag Protein Purification Kit (P2247, Beyotime). For the GST pull-down assay, GST-SOX9, His-USP28, and GST-tag Purification Resin (P2253, Beyotime) were incubated together in GST pull-down protein binding buffer for 2 h. Subsequently, the GST-tag Purification Resins were washed twice using washing buffer, boiled in SDS loading buffer, and analyzed by western blot.

### In vitro de-ubiquitination (DUB) assay

Flag-SOX9 and HA-Ub were co-transfected into HEK293T cells for 48 h. After 10 μM MG132 treatment for 6 h, IP was performed to harvest the Flag-SOX9 using an antibody against Flag-tag. His-USP28 was purified from *Escherichia coli* BL21. Then, the purified His-USP28 protein was mixed with resultant Flag-SOX9 beads in DUB assay buffer (50 mM Tris HCl pH 7.5/1 mM EDTA/100 mM NaCl/0.05% CHAPS/5 mM DTT) for 2 h at 37 °C. Finally, the mixture was boiled in SDS loading buffer and analyzed by western blot.

### Animal experiments

The animal study was ethically approved by the Animal Care and Use Committee of Qilu Hospital of Shandong University. Female BALB/c nude mice, aged 4–6 weeks, were obtained from the Gempharmatech Co., Ltd (Nanjing, China) and housed under specific pathogen-free conditions. Cells (5 × 10^6^) suspended in PBS were subcutaneously injected into the flanks of the mice. Once the tumors reached an average volume of 50 mm^3^, the mice were randomly assigned into four groups (*n* = 6 per group): (a) vehicle control, (b) AZ1, (c) olaparib, and (d) olaparib + AZ1. Then, the mice received an intraperitoneal injection of olaparib (20 mg/kg) or/and AZ1 (40 mg/kg) every day, respectively. Two weeks post-injection, the mice were sacrificed by cervical dislocation, and the xenograft tumors were excised for further analysis. The tumor volumes were calculated using the formula: length × width^2^ × 0.5.

### Comet assay

The Comet Assay was conducted using CometAssay® Silver Kit (4250-050-K, R&D Systems, MN, USA) according to the manufacturer’s protocol. In brief, treated cells were digested and resuspended at a concentration of 1 × 10^5^ cells/mL in ice-cold PBS. Cells were then mixed with molten CometAssay LMAgarose (at 37 °C) at a 1:10 (v/v) ratio and pipetted onto a CometSlide. After cell lysis and DNA unwinding, the cells were subjected to alkaline electrophoresis. The DNA was stained with SYBR Green I for 15 min in the dark. The images were captured using a fluorescent microscope (X81, Olympus Optical, Tokyo, Japan).

### Statistical analysis

The HGSOC high expression genes in the Qilu cohort (HGSOC-H) were obtained as previously described [21]. The ovarian cancer high expression genes in TCGA cohort (TCGA-H) were downloaded from GEPIA 2 (http://gepia2.cancer-pku.cn). High expression genes in olaparib-resistant SKOV3 (SKOV3/Ola) cells (PARPi-H) were acquired by comparing differentially expressed genes between SKOV3/Ola cells and parental SKOV3 cells. RNA-seq analysis was performed on SKOV3/Ola cells (*n* = 3) and SKOV3 cells (*n* = 3) by Biomarker company (Beijing, China). Data are presented as mean ± standard error of the mean (SEM). Statistical analyses were performed using the Student’s t-test for comparisons between two groups, and one-way ANOVA for comparisons among three or more groups, using GraphPad Software (La Jolla, CA, USA). All experiments were independently repeated at least three times unless otherwise indicated. *P* < 0.05 was considered as statistically significant. Spearman’s correlation coefficient was calculated using GraphPad to assess the correlation between SOX9 and USP28.

## Results

### SOX9 promotes olaparib resistance in ovarian cancer

**Fig. 1:**
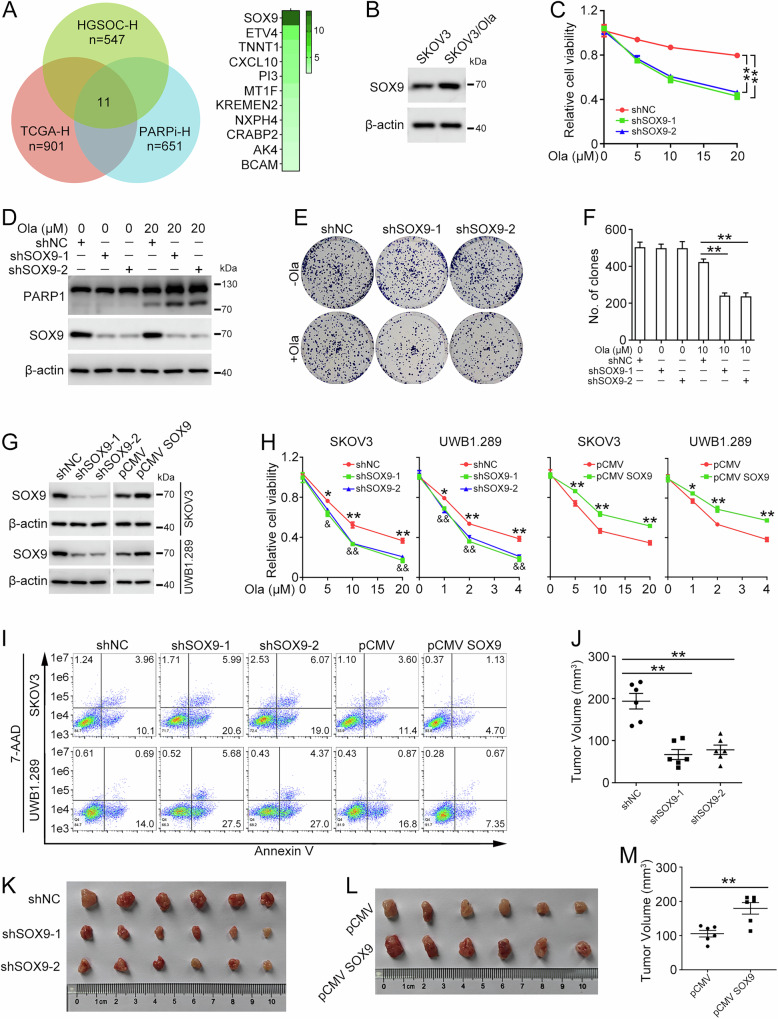
SOX9 confers resistance to olaparib in ovarian cancer. **A** Venn diagram illustrates the intersections between HGSOC high expression genes in the Qilu cohort (HGSOC-H), ovarian cancer high expression genes in TCGA cohort (TCGA-H), and high expression genes in olaparib-resistant SKOV3 (SKOV3/Ola) cells (PARPi-H). **B** Western blot was performed to determine SOX9 and β-actin protein levels in SKOV3 and SKOV3/Ola cells. Quantification of SOX9 protein level was shown in Supplementary Fig. S1A. **C** SKOV3/Ola cells were stably transfected with pLKO.1 (shNC), SOX9 shRNA 1 (shSOX9-1), and SOX9 shRNA 2 (shSOX9-2). The MTT assay was performed to detect cell viability in cells treated with olaparib (Ola; 0, 5, 10, 20 μM) for 72 h. **D** Western blot was used to determine PARP1, SOX9, and β-actin protein levels in SKOV3/Ola cells treated with 20 μM olaparib for 72 h. Quantification of PARP1 protein level was shown in Supplementary Fig. S1B. **E** Clonogenic assay was used to assess the colony formation efficiency in SKOV3/Ola cells treated with 10 μM olaparib. **F** Quantification of the number of clones in (**E**). pLKO.1 (shNC), SOX9 shRNA 1 (shSOX9-1), SOX9 shRNA 2 (shSOX9-2), pCMV, or pCMV SOX9 plasmids were stably transfected into SKOV3 and UWB1.289 cells. **G** Western blot was used to determine SOX9 and β-actin protein levels. Quantification of SOX9 protein level was shown in Supplementary Fig. S1C. **H** The MTT assay was performed to detect cell viability in cells treated with olaparib (SKOV3, 0, 5, 10, 20 μM; UWB1.289, 0, 1, 2, 4 μM) for 72 h. shSOX9-1 vs. shNC, *p < 0.05, **p < 0.01; shSOX9-2 vs. shNC, ^&^*p* < 0.05, ^& &^*p* < 0.01. **I** Flow cytometry assay was performed to detect cell apoptosis in cells treated with olaparib (SKOV3, 10 µM; UWB1.289, 2 µM) for 72 h. Quantification of the proportion of apoptotic cells was shown in Supplementary Fig. S1F. UWB1.289 cells (5 × 10^6^) stably transfected with pLKO.1 (shNC), SOX9 shRNA-1 (shSOX9-1), SOX9 shRNA-2 (shSOX9-2), pCMV, or pCMV-SOX9 were subcutaneously injected into nude mice. *n* = 6 per group. When the tumors reached an average tumor volume of 50 mm^3^, the mice received an intraperitoneal injection of olaparib (20 mg/kg) every day. Two weeks post-injection, mice were euthanized and the xenograft tumors were removed. **K,** **L** Tumor picture of each group was displayed. **J**, **M** The tumor volumes of each group in (**K**) and (**L**). (Data are presented as the mean ± SEM, *p < 0.05, **p < 0.01, *n* = 3).

To identify key factors involved in PARPi resistance, a combined analysis of HGSOC-H, TCGA-H, and PARPi-H data was performed. Among the 11 genes derived from the intersection, SOX9 exhibited the most pronounced alteration (Fig. 1A). The protein level of SOX9 in olaparib-resistant SKOV3 cells (SKOV3/Ola) was higher than that in parental SKOV3 cells (Fig. 1B). The MTT assay showed that knockdown of SOX9 dramatically increases the sensitivity of SKOV3/Ola cells to olaparib (Fig. 1C). In addition, SOX9 inhibition resulted in an increased level of cleaved PARP1 upon olaparib treatment (Fig. 1D). SOX9 knockdown also decreased the colony formation ability of SKOV3/Ola cells challenged with olaparib (Fig. 1E, F). To further investigate the function of SOX9 in the sensitivity of ovarian cancer cells to PARPi, SKOV3 and UWB1.289 cells with SOX9 knockdown or overexpression were established by lentivirus infection (Fig. 1G) and challenged with gradient concentrations of olaparib. The MTT assay showed that SOX9 knockdown sensitizes ovarian cancer cells to olaparib, whereas SOX9 overexpression led to resistance to olaparib treatment (Fig. 1H). Furthermore, flow cytometry analysis revealed a higher proportion of apoptotic cells in cells with SOX9 knockdown, whereas cells with SOX9 overexpression exhibited a decreased ratio of apoptotic cells (Fig. 1I). The findings from the colony formation assay corroborated that SOX9 fosters resistance to olaparib in ovarian cancer cells (Fig. S1D, E). Subsequently, we explored the potential role of SOX9 in conferring olaparib resistance in the xenograft mouse model. As depicted in Fig. 1J–M, overexpression of SOX9 augmented the resistance of ovarian cancer cells to olaparib, while knockdown of SOX9 exerted an opposing effect. Therefore, these data suggested that SOX9 contributes to olaparib resistance in ovarian cancer.

### PARPi promotes the interaction between SOX9 and USP28

**Fig. 2:**
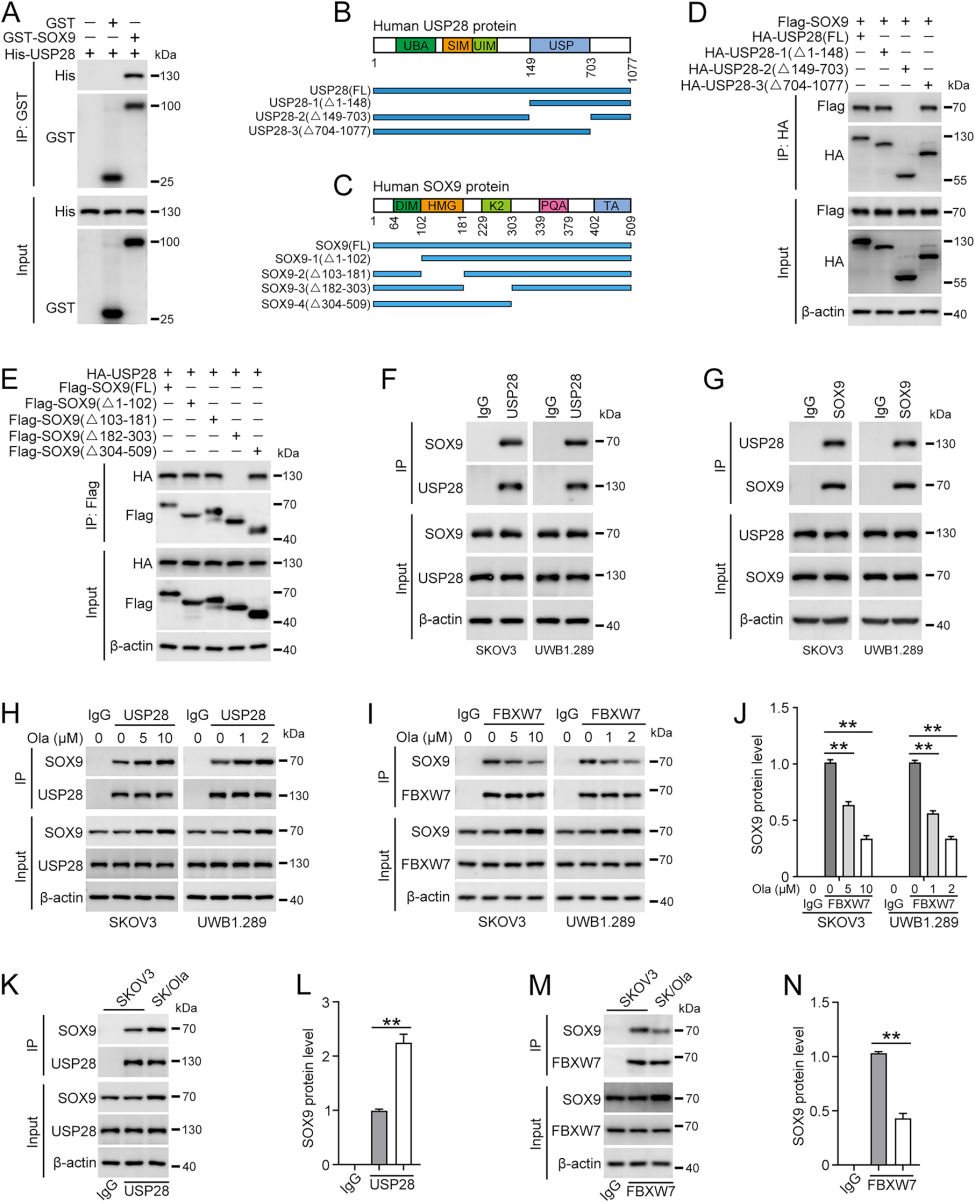
Olaparib enhances the interaction between USP28 and SOX9. **A** GST pull-down assay was used to validate the direct interaction between SOX9 and USP28. **B** The schematic diagram illustrates the full-length and truncation mutants of USP28 proteins. **C** The schematic diagram illustrates the full-length and truncation mutants of SOX9 proteins. **D** Full-length and truncated HA-tagged USP28 were co-transfected into HEK293T cells along with Flag-SOX9. Co-IP assay was performed using anti-HA beads and co-eluted SOX9 was detected using anti-Flag antibody. **E** Full-length and truncated Flag-tagged SOX9 were co-transfected into HEK293T cells along with HA-USP28. Co-IP assay was performed using anti-Flag beads and co-eluted USP28 was detected using anti-HA antibody. **F** and **G** Co-IP was performed to verify the endogenous interaction between SOX9 and USP28. **H** Cells were treated with olaparib (Ola) for 24 h, followed by pulldown with anti-USP28 antibody and immunoblotting with the antibodies indicated. Quantification of the IPed SOX9 protein level was shown in Supplementary Fig. S2C. **I** Cells were treated with olaparib (Ola) for 24 h, followed by pulldown with anti-FBXW7 antibody and immunoblotting with the antibodies indicated. **J** Quantification of the IPed SOX9 protein level in (**I**). **K** Western blot was used to detect the SOX9 Co-IPed with anti-USP28 antibody in SKOV3 and SKOV3/Ola cells. **L** Quantification of the IPed SOX9 protein level in (**K**). **M** Western blot was used to detect the SOX9 Co-IPed with anti-FBXW7 antibody in SKOV3 and SKOV3/Ola cells. **N** Quantification of the IPed SOX9 protein level in (**M**). (Data are presented as the mean ± SEM, *p < 0.05, **p < 0.01, *n* = 3).

As inhibitors specifically targeting SOX9 are not yet available, we focused on elucidating the mechanisms driving its upregulation in ovarian cancer cells upon olaparib treatment. A mass spectrometry-based proteomic analysis revealed USP28 as a novel interaction partner of SOX9 (Fig. S2A). The GST pull-down assay was implemented to validate the direct interaction between SOX9 and USP28 (Fig. 2A). To identify the specific domains of USP28 that mediate this interaction, various truncation mutants of USP28 were generated and evaluated for their ability to interact with SOX9. Different regions of USP28 were HA-tagged, co-expressed with Flag-SOX9, and immunoprecipitated with anti-HA beads. As illustrated in Fig. 2B, D, the USP domain of USP28 is indispensable for its binding with SOX9, while other domains are not essential for this interaction. We further mapped the region in SOX9 that could mediate the interaction. It is apparent that the K2 domain of SOX9 is required for interacting with USP28 (Fig. 2C, E). The endogenous interaction between USP28 and SOX9 was confirmed in SKOV3 and UWB1.289 cells (Fig. 2F, G). In contrast, antibodies against USP25, a ubiquitin-specific protease that shares an identical domain structure with USP28, did not co-immunoprecipitate SOX9, nor did antibodies against SOX9 co-immunoprecipitate USP25 (Fig. S2B). To explore the impact of PARPi on the interaction between USP28 and SOX9, cells were treated with olaparib for 48 h. The Co-IP results showed that olaparib treatment enhances the binding between USP28 and SOX9 (Fig. 2H). It has been reported that the E3 ubiquitin ligase F-box and WD repeat domain-containing 7 (FBXW7) interact with SOX9, facilitating its degradation through ubiquitin-dependent proteolysis [22, 23]. Meanwhile, the activity of FBXW7 can be antagonized by USP28, which binds directly to FBXW7 [24, 25]. Therefore, we investigated the effect of PARPi on the interaction between SOX9 and FBXW7. Our results indicated that olaparib treatment disrupts the binding between SOX9 and FBXW7 (Fig. 2I, J). Additionally, we observed enhanced binding between USP28 and SOX9, but weakened binding between FBXW7 and SOX9 in SKOV3/Ola cells (Fig. 2K–N). In conclusion, these data indicated a direct interaction between USP28 and SOX9, suggesting a potential role of USP28 in the regulation of SOX9 ubiquitination.

### USP28 regulates SOX9 protein stability

**Fig. 3:**
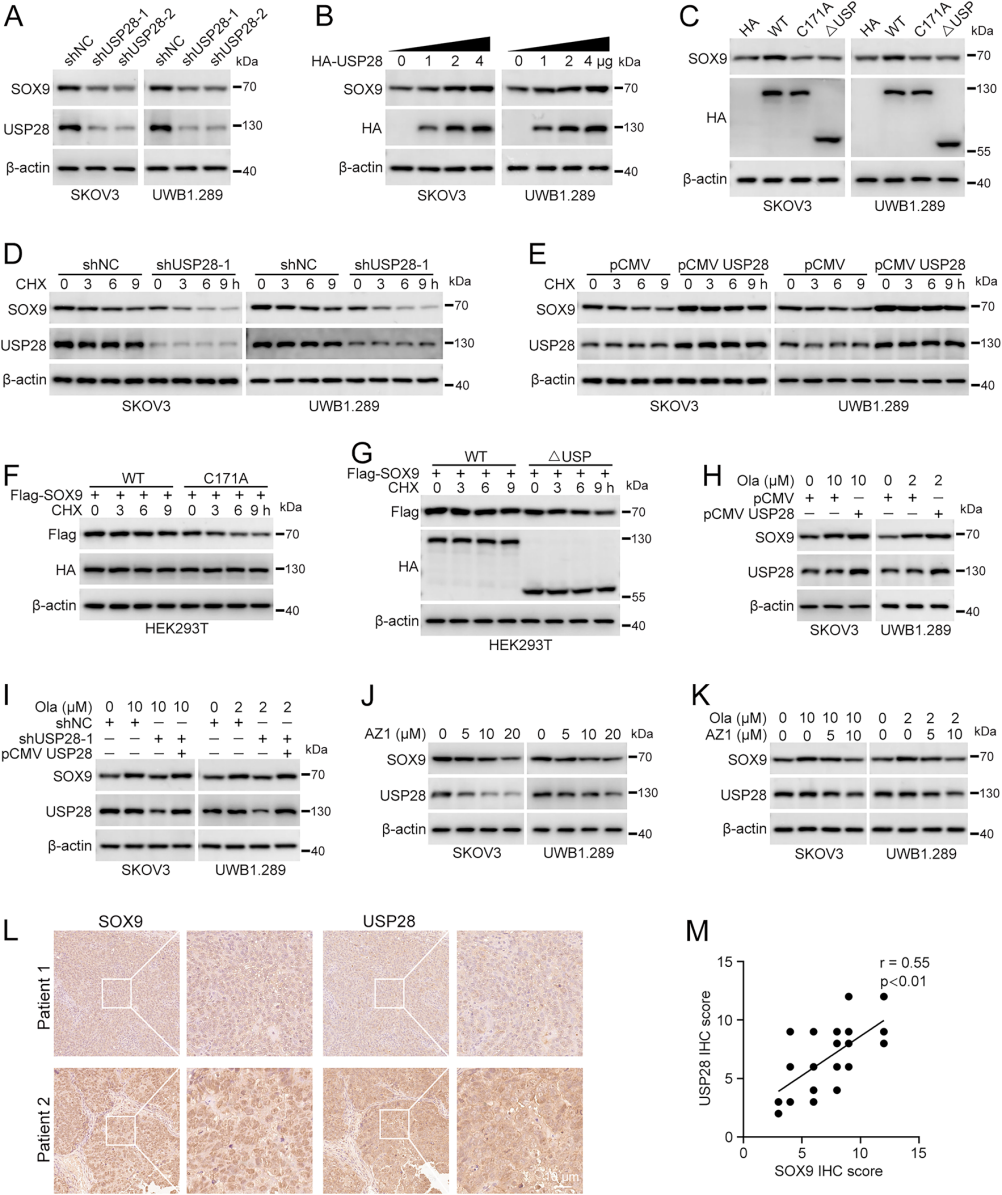
USP28 controls SOX9 protein stability. **A** Western blot was performed to detect the protein levels of SOX9 in cells with USP28 knockdown. **B** HA-USP28 (0, 1, 2, 4 µg) were transiently transfected into cells for 48 h. The protein levels of SOX9 and HA-USP28 were detected using western blot. **C** Empty vector (HA), HA-USP28 (WT), HA-USP28^C171A^ (C171A), and HA-USP28^ΔUSP^ (ΔUSP) were transiently transfected into cells for 48 h. The protein levels of SOX9 and HA-USP28 were analyzed using western blot. **D** Half-life analysis of SOX9 protein in cells with USP28 knockdown. Cells were treated with cycloheximide (CHX, 100 μg/ml) for the indicated time points. **E** Half-life analysis of SOX9 protein in cells with USP28 overexpression. Cells were treated with 100 μg/ml CHX for the indicated time points. **F** Half-life analysis of Flag-SOX9 protein in HEK293T cells transfected with HA-USP28 (WT) and HA-USP28^C171A^ (C171A). Cells were treated with 100 μg/ml CHX for the indicated time points. **G** Half-life analysis of Flag-SOX9 protein in HEK293T cells transfected with HA-USP28 (WT) and HA-USP28^ΔUSP^ (ΔUSP). Cells were treated with 100 μg/ml CHX for the indicated time points. **H** Cells stably transfected with pCMV or pCMV-USP28 were treated with olaparib for 48 h. The protein levels of SOX9 and USP28 were detected using western blot. **I** Cells stably transfected with pLKO.1 (shNC), USP28 shRNA 1 (shUSP28-1), and/or pCMV-USP28 were treated with olaparib for 48 h. The protein levels of SOX9 and USP28 were detected using western blot. **J** Cells were treated with gradient concentrations of AZ1 for 48 h. The protein levels of SOX9 and USP28 were detected using western blot. **K** Cells were treated with different concentrations of AZ1 and/or olaparib for 48 h. The protein levels of SOX9 and USP28 were detected using a western blot. **L** Representative images of IHC staining of SOX9 and USP28 in HGSOC tissues. Scale bar: 10 µm. A total of 1000 cells per group were counted to calculate the staining intensity. **M** Correlation between SOX9 and USP28 expression in HGSOC patients. Quantification of protein levels in (**A**–**K**) was shown in Supplementary Fig. S3H–R. (Data are presented as the mean ± SEM, *p < 0.05, **p < 0.01, *n* = 3).

Firstly, we investigated whether USP28 can modulate the expression level of SOX9. As expected, knockdown of USP28 resulted in the downregulation of SOX9 protein level (Fig. 3A), with no impact on its mRNA level (Fig. S3A). In contrast, the overexpression of USP28 increased both endogenous and exogenous protein levels of SOX9 in a dose-dependent manner (Fig. 3B and Fig. S3B, C). However, the catalytically inactive form (USP28^C171A^) or the ΔUSP truncation mutant of USP28 (USP28^ΔUSP^) failed to enhance the expression of SOX9 (Fig. 3C). In addition, the downregulation of SOX9 protein level induced by USP28 knockdown could be restored by reconstitution of USP28 (Fig. S3D, E). MG132, a proteasome inhibitor, prevented the downregulation of SOX9 in USP28-depleted cells, indicating that USP28 maintains the SOX9 stability through its function as a deubiquitinase (Fig. S3F, G). Next, we proceeded to evaluate the influence of USP28 on the half-life of SOX9. USP28 knockdown shortened the half-life of SOX9 (Fig. 3D), whereas overexpression of WT USP28, but not the inactive form or ΔUSP truncation, prolonged the half-life of SOX9 (Fig. 3E–G). Subsequently, we investigated the effect of USP28 on SOX9 protein level in the presence of olaparib. Upon olaparib treatment, SOX9 protein level was increased in cells with USP28 overexpression, but decreased in cells with USP28 knockdown. This decrease could be reversed by reintroducing USP28 (Fig. 3H, I). The pharmacological inhibitor of USP28, AZ1, decreased the protein levels of USP28 itself and that of SOX9 in a dose-dependent manner (Fig. 3J). Additionally, AZ1 suppressed the olaparib-induced upregulation of SOX9 protein levels (Fig. 3K). We further performed IHC staining for USP28 and SOX9 in HGSOC tissues from our cohort. The results demonstrated a positive correlation between the protein levels of USP28 and SOX9 (Fig. 3L, M). Whereas, knockdown of USP25 did not affect SOX9 protein level. Meanwhile, AZ1 was still able to reduce the protein level of SOX9 in cells with USP25 knockdown (Fig. S4A, B). In conclusion, these findings suggested that USP28 plays a role in maintaining the stability of SOX9 in ovarian cancer.

### USP28 mediates the ubiquitination of SOX9 as a deubiquitinase

**Fig. 4:**
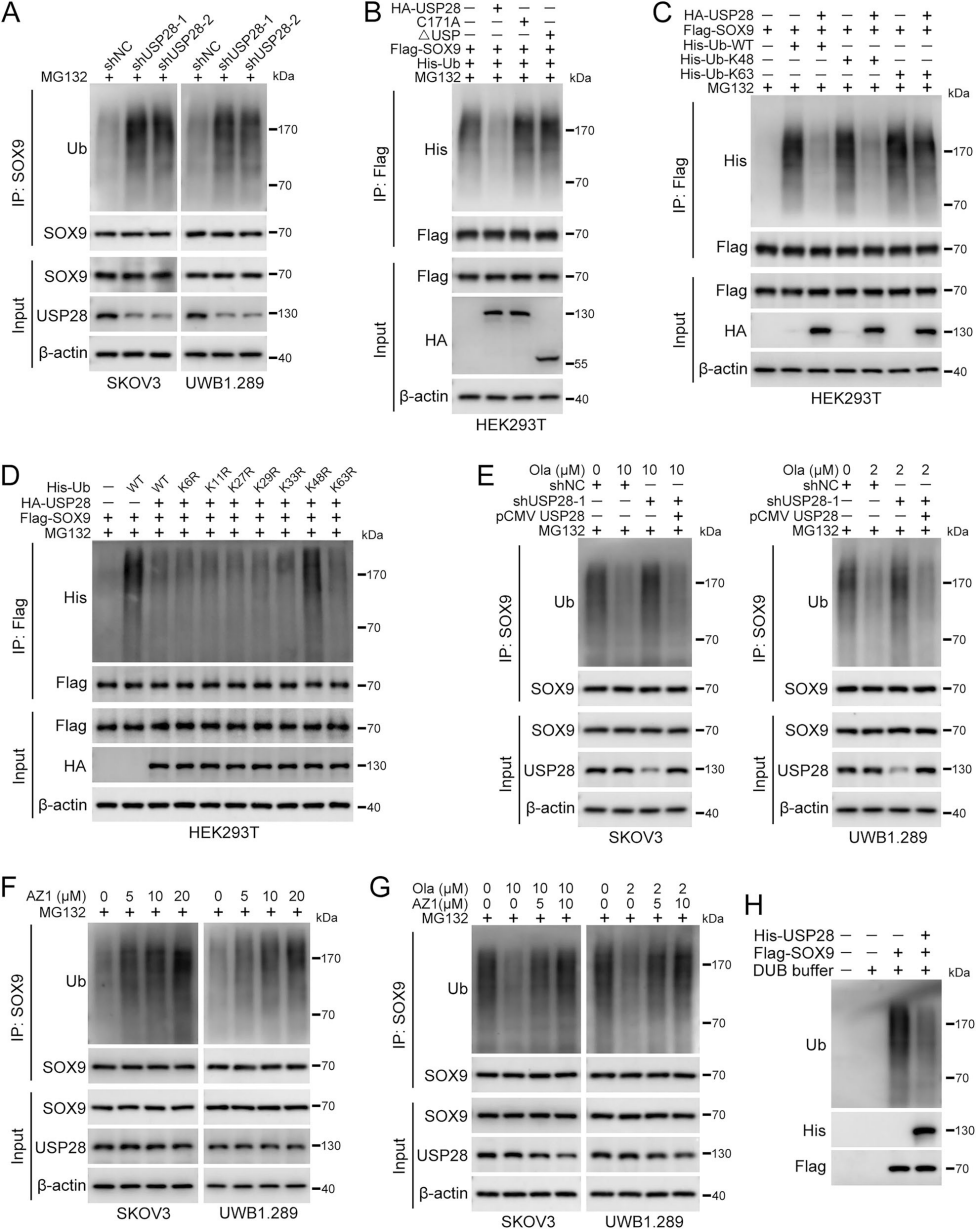
USP28 mediates ubiquitination of SOX9. **A** The ubiquitination assay of SOX9 in cells with USP28 knockdown. Co-IP was performed with anti-SOX9 antibody and immunoblotted with the antibodies indicated. Cells were treated with MG132 (10 μM) for 6 h before sample collection. **B** HEK293T cells were transfected with HA-USP28, HA-USP28^C171A^ (C171A), HA-USP28^ΔUSP^ (ΔUSP), Flag-SOX9, and His-Ub for 48 h. MG132 (10 μM) was added 6 h before harvest. Co-IP was performed with anti-Flag beads and immunoblotted with the antibodies indicated. **C** HEK293T cells were transfected with HA-USP28, Flag-SOX9, His-Ub-WT, His-Ub-K48, and His-Ub-K63 for 48 h. MG132 (10 μM) was added 6 h before harvest. Co-IP was performed with anti-Flag beads and immunoblotted with the antibodies indicated. **D** HEK293T cells were transfected with HA-USP28, Flag-SOX9, His-Ub (WT, K6R, K11R, K27R, K29R, K33R, K48R, or K63R) for 48 h. MG132 (10 μM) was added 6 h before harvest. Co-IP was performed with anti-Flag beads and immunoblotted with the antibodies indicated. **E** Cells stably transfected with pLKO.1 (shNC), USP28 shRNA 1 (shUSP28-1), and/or pCMV-USP28 were treated with olaparib for 48 h. MG132 (10 μM) was added 6 h before harvest. Co-IP was performed with anti-SOX9 antibody and immunoblotted with the antibodies indicated. **F** Cells were treated with AZ1 for 48 h. MG132 (10 μM) was added 6 h before harvest. Co-IP was performed with anti-SOX9 antibody and immunoblotted with the antibodies indicated. **G** Cells were treated with AZ1 and/or olaparib for 48 h. MG132 (10 μM) was added 6 h before harvest. Co-IP was performed with anti-SOX9 antibody and immunoblotted with the antibodies indicated. **H** In vitro de-ubiquitination analysis of USP28. Flag-SOX9 and HA-Ub were co-transfected into HEK293T cells for 48 h. After 10 μM MG132 treatment for 6 h, Flag-SOX9 (including ubiquitinated form) was pulled down and incubated with purified His-USP28. Quantification of protein levels in (**A**–**G**) was shown in Supplementary Fig. S6A–G.

We further investigated whether USP28 acts as a deubiquitinase for SOX9. As shown in Fig. 4A, knockdown of USP28 increased the levels of ubiquitin in SOX9 immunoprecipitates. In contrast, overexpression of USP28, but not its inactive form or ΔUSP truncation, led to decreased ubiquitination of SOX9 (Fig. 4B), suggesting that the catalytic activity of USP28 is essential for SOX9 deubiquitination. To identify the type of polyubiquitin chains removed by USP28, we co-transfected cells with His-Ub (WT, K48-only, or K63-only), HA-USP28, and Flag-SOX9. Our observations indicated that USP28 specifically reduces K48-linked but not K63-linked ubiquitination of SOX9 (Fig. 4C). Moreover, overexpression of USP28 failed to remove the ubiquitination of SOX9 in cells transfected with K48R (mutation of only Lys48 to Arg) mutant (Fig. 4D). We then examined whether FBXW7 is involved in USP28-mediated SOX9 deubiquitination. Expression of wild-type FBXW7, but not its catalytically inactive form (FBXW7^R465A^), decreased protein level and increased ubiquitination of SOX9, and these effects were reversed by expressing WT USP28 (Fig. S5A, B). Consistent with our previous findings that olaparib enhances the interaction between USP28 and SOX9, we also demonstrated that olaparib treatment reduces SOX9 ubiquitination. Knockdown of USP28 abolished this olaparib-induced effect, which was restored by re-expression of USP28 (Fig. 4E). In addition, USP28 specific inhibitor AZ1 dose-dependently increased the ubiquitination of SOX9 and mitigated the decrease in SOX9 ubiquitination induced by olaparib treatment (Fig. 4F, G). The in vitro de-ubiquitination assay also showed that purified USP28 effectively removes ubiquitination from SOX9 (Fig. 4H). Collectively, our results revealed that USP28 prevents SOX9 proteasomal degradation by eliminating K48-linked polyubiquitin chains.

### USP28 promotes olaparib resistance through SOX9

**Fig. 5:**
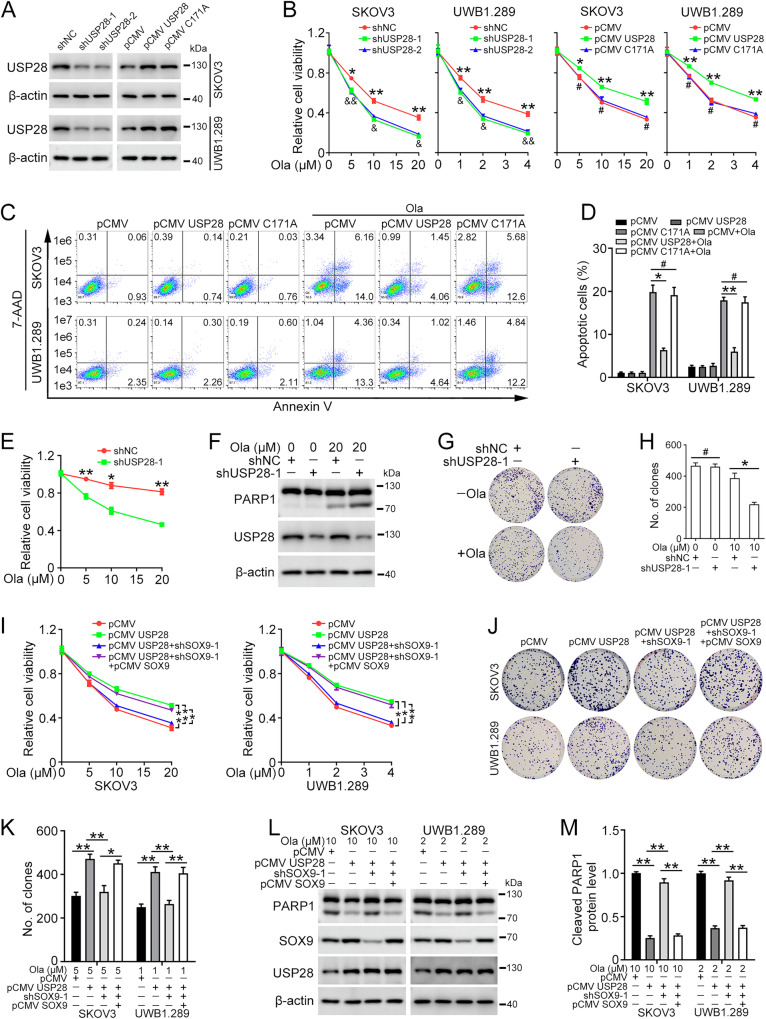
USP28 promotes olaparib resistance through interacting with SOX9. pLKO.1 (shNC), USP28 shRNA 1 (shUSP28-1), USP28 shRNA 2 (shUSP28-2), pCMV, pCMV USP28, or pCMV USP28^C171A^ (pCMV C171A) plasmids were stably transfected into SKOV3 and UWB1.289 cells. **A** Western blot was used to determine USP28 protein levels. Quantification of USP28 protein level was shown in Supplementary Fig. S7E. **B** The MTT assay was performed to detect cell viability in cells treated with olaparib (SKOV3, 0, 5, 10, 20 μM; UWB1.289, 0, 1, 2, 4 μM) for 72 h. shUSP28-1 vs. shNC, *p < 0.05, **p < 0.01; shUSP28-2 vs. shNC, ^&^*p* < 0.05, ^& &^*p* < 0.01; pCMV USP28 vs. pCMV, *p < 0.05, **p < 0.01; pCMV vs. pCMV C171A, ^#^*p* > 0.05. **C** Flow cytometry assay was performed to detect cell apoptosis in cells treated with olaparib (SKOV3, 10 µM; UWB1.289, 2 µM) for 72 h. **D** Quantification of the proportion of apoptotic cells in (**C**). **E** SKOV3/Ola cells were stably transfected with pLKO.1 (shNC) and USP28 shRNA 1 (shUSP28-1). The MTT assay was performed to detect cell viability in cells treated with olaparib (0, 5, 10, 20 μM) for 72 h. **F** Western blot was used to determine PARP1 and USP28 protein levels in SKOV3/Ola cells treated with 20 μM olaparib for 72 h. Quantification of PARP1 protein level was shown in Supplementary Fig. S7F. **G** Clonogenic assay was used to assess the colony formation efficiency in SKOV3/Ola cells treated with 10 μM olaparib. **H** Quantification of the number of clones in (**G**). pCMV, pCMV USP28, SOX9 shRNA 1 (shSOX9-1), and/or pCMV SOX9 plasmids were stably transfected into SKOV3 and UWB1.289 cells. **I** The MTT assay was performed to detect cell viability in cells treated with olaparib for 72 h. **J** Clonogenic assay was used to assess the colony formation efficiency of cells treated with olaparib (SKOV3, 5 µM; UWB1.289, 1 µM). **K** Quantification of the number of clones in (**J**). **L** Western blot was used to determine PARP1, SOX9, and USP28 protein levels in cells treated with olaparib for 72 h. **M** Quantification of PARP1 protein level in (**L**). (Data are presented as the mean ± SEM, *p < 0.05, **p < 0.01, *n* = 3).

To further elucidate the role of USP28 in modulating the sensitivity of ovarian cancer cells to olaparib, we constructed lentivirus-infected ovarian cancer cells with either USP28 knockdown or overexpression, followed by treatment with gradient concentrations of olaparib (Fig. 5A). Results from the MTT assay demonstrated that USP28 knockdown heightens the sensitivity of ovarian cancer cells to olaparib. Conversely, overexpression of WT USP28, but not the inactive form (USP28^C171A^), conferred resistance to olaparib (Fig. 5B). Flow cytometry analysis (Fig. 5C, D), colony formation assay (Fig. S7A, B), and western blot (Fig. S7C, D) yielded the similar conclusions that WT USP28 enhances ovarian cancer cells resistance to olaparib, while USP28^C171A^ had no apparent effects.

Next, we delineated the impact of USP28 on olaparib sensitivity in SKOV3/Ola cells. It was observed that SKOV3/Ola cells exhibit minimal response to olaparib at concentrations of 5 µM and 10 µM. However, upon knockdown of USP28, these cells regained sensitivity to olaparib (Fig. 5E). In addition, the expression of cleaved PARP1 induced by olaparib was notably increased in cells with USP28 knockdown compared to those in the control group (Fig. 5F). Moreover, USP28 knockdown also attenuated the colony formation ability of SKOV3/Ola cells upon exposure to olaparib (Fig. 5G, H).

We further investigated whether USP28-mediated olaparib resistance depends on SOX9 expression. Indeed, the MTT assay showed that SOX9 knockdown impedes the effect of USP28 overexpression, which could be abolished by reconstruction of SOX9 expression (Fig. 5I). The colony formation experiments affirmed that the olaparib resistance induced by USP28 overexpression could be mitigated by SOX9 knockdown, and further reversed upon the restoration of SOX9 expression (Fig. 5J, K). The effect of USP28/SOX9 was verified by detecting apoptotic proteins using western blot (Fig. 5L, M). Thus, our data indicated that USP28 cooperates with SOX9 to promote resistance to olaparib in ovarian cancer.

### SOX9 directly binds and regulates DDR gene

**Fig. 6:**
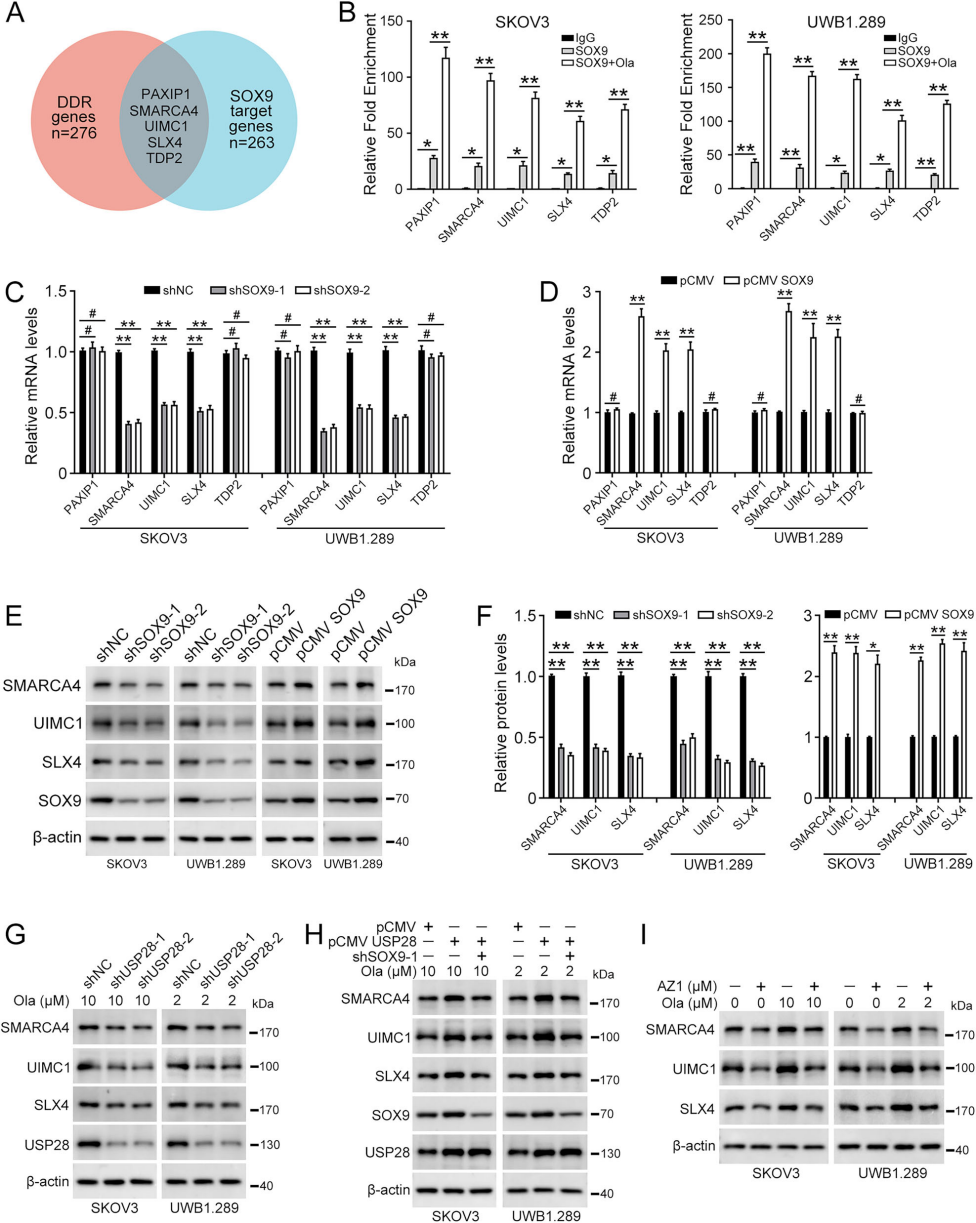
SOX9 directly binds the DDR gene promoters and induces their expression. **A** Venn diagram illustrates the intersections between core DDR genes (*n* = 276) and SOX9 target genes (*n* = 263) identified by ChIP-Seq. **B** Cells were treated with or without olaparib (SKOV3, 10 µM; UWB1.289, 2 µM) for 48 h. qPCR analysis of ChIP samples from experiments performed in SKOV3 and UWB1.289 cells using the anti-SOX9 antibody or IgG. **C** qPCR was performed to detect the mRNA levels of *PAXIP1*, *SMARCA4*, *UIMC1*, *SLX4*, and *TDP2* in cells stably transfected with pLKO.1 (shNC), SOX9 shRNA 1 (shSOX9-1), or SOX9 shRNA 2 (shSOX9-2). Cells were treated with olaparib (SKOV3, 10 µM; UWB1.289, 2 µM) for 48 h before harvest. **D** qPCR was performed to detect the mRNA levels in cells stably transfected with pCMV or pCMV SOX9. Cells were treated with olaparib (SKOV3, 10 µM; UWB1.289, 2 µM) for 48 h before harvest. **E** Western blot was performed to detect the protein levels of SMARCA4, UIMC1, SLX4, and SOX9 in cells stably transfected with pLKO.1 (shNC), SOX9 shRNA 1 (shSOX9-1), SOX9 shRNA 2 (shSOX9-2), pCMV, or pCMV SOX9. Cells were treated with olaparib (SKOV3, 10 µM; UWB1.289, 2 µM) for 48 h before harvest. **F** Quantification of the protein levels in (**E**). **G** Cells transfected with pLKO.1 (shNC), USP28 shRNA 1 (shUSP28-1), or USP28 shRNA 2 (shUSP28-2) were challenged with olaparib for 48 h, and the protein levels of SMARCA4, UIMC1, SLX4, USP28, and SOX9 were detected using western blot. **H** Cells transfected with pCMV, pCMV USP28, and SOX9 shRNA 1 (shSOX9-1) were challenged with olaparib for 48 h, and the protein levels were detected using western blot. **I** Cells were treated with AZ1 (10 µM) and/or olaparib for 48 h. Western blot was used to detect protein levels. Quantification of protein levels in Fig. 6G–I was shown in Supplementary Fig. S9B–D. (Data are presented as the mean ± SEM, *p < 0.05, **p < 0.01, *n* = 3).

We then turned to explore the mechanism by which SOX9 regulates PARPi resistance in ovarian cancer. ChIP-Seq was conducted to identify SOX9 binding peaks in UWB1.289 cells. We obtained 263 binding peaks of SOX9, with 80% of them distributed in the promoter region. Then, we intersected the genes bound by SOX9 with 276 key DNA Damage Repair (DDR) genes [26], resulting in five genes: *PAXIP1*, *SMARCA4*, *UIMC1*, *SLX4*, and *TDP2A* (Fig. 6A and Fig. S8). Subsequently, ChIP-PCR assays revealed that SOX9 directly binds to the promoter regions of these genes, and the bindings were enhanced upon olaparib treatment (Fig. 6B). The qRT-PCR showed that knockdown of SOX9 decreases, and overexpression of SOX9 increases the mRNA levels of *SMARCA4*, *UIMC1*, and *SLX4* following olaparib treatment. In contrast, either knockdown or overexpression of SOX9 had no effect on the mRNA levels of *PAXIP1* and *TDP2A* (Fig. 6C, D). Western blot assay also revealed that overexpression of SOX9 elevates the protein levels of these three factors, while knockdown of SOX9 decreases their protein levels in the presence of olaparib (Fig. 6E, F). Next, we investigated whether USP28 regulates the expression of these three DDR genes by interacting with SOX9. Knockdown of USP28 reduced the binding of SOX9 to the promoter regions of *SMARCA4*, *UIMC1*, and *SLX4* (Fig. S9A). Besides, downregulation of USP28 led to a reduction in the protein levels of SMARCA4, UIMC1, and SLX4 (Fig. 6G and Fig. S9B). Overexpression of USP28 enhances the expression of these three proteins, whereas knockdown of SOX9 counteracts this effect (Fig. 6H and Fig. S9C). We observed that olaparib promotes the expression of SMARCA4, UIMC1, and SLX4 in ovarian cancer cells. Additionally, under both conditions with or without olaparib treatment, USP28 inhibitor AZ1 prominently reduces the expression levels of SMARCA4, UIMC1, and SLX4. (Fig. 6I and Fig. S9D). In conclusion, these findings suggested that USP28/SOX9 positively regulates the expression of DDR genes (*SMARCA4*, *UIMC1*, and *SLX4*) by binding to their promoters in ovarian cancer cells.

### USP28/SOX9 alleviates the DNA damage induced by olaparib

**Fig. 7:**
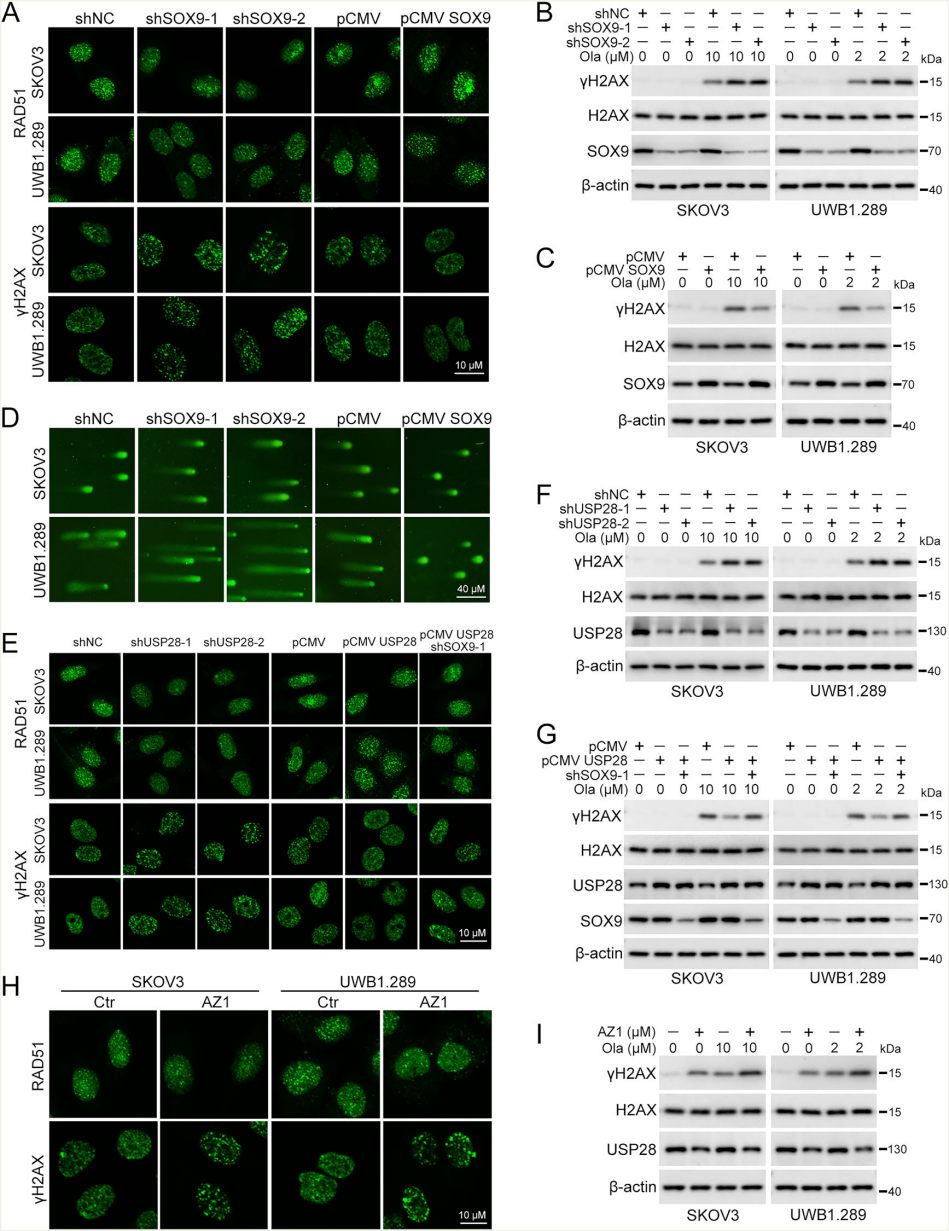
USP28/SOX9 regulates DNA damage repair upon olaparib treatment. Cells transfected with pLKO.1 (shNC), SOX9 shRNA 1 (shSOX9-1), SOX9 shRNA 2 (shSOX9-2), pCMV, or pCMV SOX9 were treated with olaparib (SKOV3, 10 µM; UWB1.289, 2 µM) for 48 h. **A** RAD51 and γH2AX foci were examined by immunofluorescence staining. Scale bar: 10 µm. **B**, **C** The protein levels of γH2AX, H2AX, and SOX9 were detected using western blot. **D** The Comet Assay was performed to evaluate DNA damage. Scale bar: 40 µm. Cells transfected with pLKO.1 (shNC), USP28 shRNA 1 (shUSP28-1), USP28 shRNA 2 (shUSP28-2), pCMV, pCMV USP28, and/or SOX9 shRNA 1 (shSOX9-1) were treated with olaparib (SKOV3, 10 µM; UWB1.289, 2 µM) for 48 h. **E** RAD51 and γH2AX foci were examined by immunofluorescence staining. Scale bar: 10 µm. **F**, **G** The protein levels of γH2AX, H2AX, USP28, and SOX9 were detected using western blot. Cells were treated with AZ1 (10 µM) and/or olaparib for 48 h. **H** RAD51 and γH2AX foci were examined by immunofluorescence staining. Scale bar: 10 µm. **I** The protein levels of γH2AX, H2AX, and USP28 were detected using western blot. Quantification of Fig. 7A–I was shown in Supplementary Fig. S10A–I (Data are presented as the mean ± SEM, *p < 0.05, **p < 0.01, *n* = 3).

Considering the regulatory role of USP28/SOX9 in key DDR gene expression, we then explore their effects in olaparib-induced DDR. γ-H2AX serves as a molecular marker for DNA double-strand breaks (DSBs), while RAD51 plays a crucial role in the process of homologous recombination (HR) during double-strand break repair [27, 28]. IF staining showed that knockdown of SOX9 resulted in a reduction in RAD51 foci accumulation and a concomitant increase in γH2AX foci accumulation when compared with the control group following olaparib treatment, indicative of aggravated DNA damage and defective DNA repair capability in the SOX9 deficient setting of ovarian cancer cells. However, overexpression of SOX9 exhibited completely opposite results (Fig. 7A). Consistent with these findings, SOX9 knockdown decreased the protein level of γH2AX in response to olaparib, while SOX9 overexpression had the opposite effect (Fig. 7B, C). The Comet Assay further corroborated these results, showing that SOX9 knockdown exacerbates DNA damage, while SOX9 overexpression confers a protective effect on the cells (Fig. 7D). In addition, both USP28 knockdown and AZ1 treatment decreased RAD51 accumulation and increased γH2AX accumulation following olaparib treatment in SKOV3 and UWB1.289 cells. Conversely, USP28 overexpression elicited the opposite effects, which could be abolished by suppressing SOX9 (Fig. 7E, H). Western blot analysis of γH2AX came to the same conclusions, showing that inhibition of USP28 induces DNA damage (Fig. 7F, I), while overexpression of USP28 led to an alleviated DNA damage upon olaparib treatment (Fig. 7G). Therefore, these data demonstrated that targeting USP28/SOX9 triggers DNA double-strand break damage and hampers homologous recombination repair in ovarian cancer cells.

### USP28 inhibitor sensitizes ovarian cancer to olaparib

**Fig. 8:**
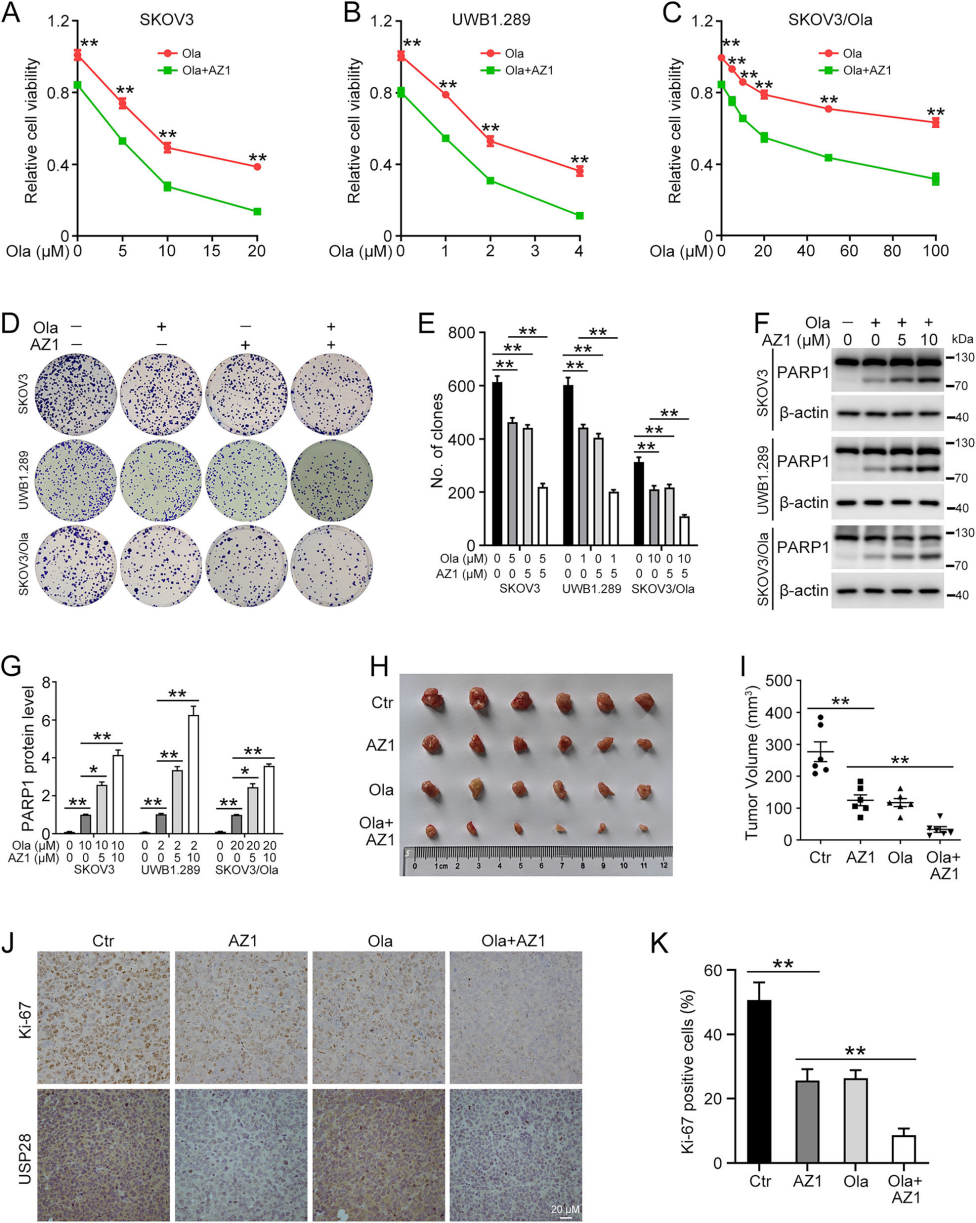
USP28 inhibitor AZ1 sensitizes cells to olaparib treatment. **A**–**C** The MTT assay was performed to determine the cell viability in SKOV3, UWB1.289, and SKOV3/Ola cells treated with AZ1 (10 µM) and gradient concentrations of olaparib for 72 h. **D** Clonogenic assay was used to assess the colony formation efficiency in cells treated with AZ1 and/or olaparib. **E** Quantification of the number of clones in (**D**). **F** Western blot was used to determine PARP1 protein levels in cells treated with AZ1 and/or olaparib (SKOV3, 10 µM; UWB1.289, 2 µM; SKOV3/Ola, 20 µM) for 72 h. **G** Quantification of PARP1 protein levels in (**F**). **H** Photos of subcutaneous tumors. **I** The tumor volumes were computed. **J** Representative photographs of IHC staining of Ki-67 and USP28 in xenograft tumors. **K** Quantification of the positive ratio of Ki-67 in (**J**). Scale bar: 20 µm (Data are presented as the mean ± SEM, *p < 0.05, **p < 0.01, *n* = 3).

To evaluate the synergistic effect of USP28 inhibitor and olaparib, gradient concentrations of olaparib were administered to SKOV3 and UWB1.289 cells along with AZ1. The MTT assay revealed an enhancement in the anti-tumor efficacy of olaparib upon AZ1 application (Fig. 8A, B). USP28 inhibition using AZ1 also notably sensitized SKOV3/Ola cells to olaparib treatment (Fig. 8C). Colony formation assays demonstrated that the combination of AZ1 and olaparib markedly reduced the number of colonies compared to either agent alone (Fig. 8D, E). Furthermore, AZ1 increased the level of apoptosis induced by olaparib treatment (Fig. 8F, G). To validate our in vitro findings, we conducted in vivo investigations into the effects of AZ1 in combination with olaparib. UWB1.289 cells were subcutaneously injected into nude mice, followed by treatment with single agents (AZ1 or olaparib) or the combination. Intraperitoneal injection of either AZ1 or olaparib alone led to decreased tumor volumes compared to the control group. Importantly, the combination treatment showed greater inhibition of tumor growth than either agent alone (Fig. 8H, I). In addition, IHC analysis of Ki-67 and USP28 corroborated the in vitro findings (Fig. 8J, K). Collectively, the use of a USP28 inhibitor in conjunction with olaparib showed promising potential in overcoming PARPi resistance in ovarian cancer.

## Discussion

SOX9 protein stability is tightly regulated through ubiquitination-dependent proteasomal degradation mechanisms. Maintaining precise control over SOX9 protein levels is crucial, as even minor increases can significantly impact tumor initiation and progression. FBXW7 is a prominent E3 ubiquitin ligase responsible for governing the ubiquitination and degradation of SOX9. GSK3β-mediated phosphorylation of SOX9 enhances its interaction with FBXW7, leading to its degradation [22, 23]. KEAP1 has also been identified as an E3 ligase for SOX9. Mutations in KEAP1 impede its ubiquitin ligase activity toward SOX9, promoting cancer cell growth and tumorigenesis by stabilizing SOX9 [29]. Beyond E3 enzyme-mediated ubiquitination, the deubiquitination and stability of SOX9 are also regulated by the deubiquitinase USP18 in reactive astrogliosis [30]. Asymmetric dimethylarginine (ADMA) exerts a dual function by interacting with both SOX9 and its associated deubiquitinating enzyme, USP7. This interaction disrupts USP7-mediated deubiquitination and leads to SOX9 degradation in osteoarthritis [31]. However, the regulation of SOX9 stability via ubiquitin modification in ovarian cancer remains poorly understood. In the current study, we performed IP/MS and, for the first time, identified deubiquitinase USP28 as a novel SOX9-interacting protein in ovarian cancer. The direct interaction between these proteins relies on the USP domain of USP28 and the K2 domain of SOX9. Overexpression of WT USP28, but not its catalytically inactive form (USP28^C171A^) or the ΔUSP truncation mutant (USP28^ΔUSP^), induced SOX9 deubiquitination and inhibited its degradation. Thus, our investigation provides further insight into the ubiquitination-based regulatory mechanism governing SOX9 protein levels.

The process of protein ubiquitination is reversible, facilitated by deubiquitinating enzymes (DUBs), which cleave the bond between ubiquitin and target proteins. The USP family is the largest group among the DUBs. USP28, a member of this family, exerts its influence on a wide range of substrates, such as TCF7L2 [32], c-MYC [33–36], SREBP2 [37], ∆Np63 [38, 39], c-JUN [36], NICD1 [36]. By regulating these substrates, USP28 contributes to various processes of cancer, such as tumorigenesis, cell proliferation, DNA damage repair, and chemotherapy resistance. Studies have shown that USP28 can reverse ubiquitination induced by several E3 ligases, with FBXW7 being particularly notable [40]. FBXW7, part of the F-box family of E3 ligases, is a key component of the Skp1-Cdc53/Cullin-F-box (SCF) protein complex [41, 42]. USP28 counteracts FBXW7 to regulate the stability of crucial oncogenic proteins like c-MYC, c-JUN, NOTCH1, and ΔNp63 [33, 38, 43]. Several studies have indicated that FBXW7 modulates the ubiquitination of SOX9 protein [22, 23, 44]. However, the functional significance of the USP28-FBXW7 partnership in maintaining SOX9 stability remains unexplored. In this study, we demonstrated that FBXW7 can induce the ubiquitination and degradation of SOX9, while USP28 opposes the function of FBXW7, thereby stabilizing SOX9. Thus, USP28 acts as the deubiquitinase responsible for maintaining SOX9 stability and protecting it from FBXW7-mediated proteasomal degradation.

SOX9 serves as a pivotal transcription factor, exerting a multifaceted influence by governing cancer stem cells (CSCs) and epithelial-mesenchymal transition (EMT) in various cancer types. Moreover, extensive evidence underscores that SOX9 drives drug resistance by regulating pro-survival signaling pathways and stemness properties across multiple cancers [22, 29, 45–48]. SOX9 also participates in controlling DNA damage response upon chemotherapy treatments. SOX9 contributes to the development of gemcitabine resistance in intrahepatic cholangiocarcinoma by inhibiting CHK1-mediated homologous recombination repair (HRR) activity [49]. Additionally, the activation of the SOX9/MMS22L-dependent DNA damage pathway promotes oxaliplatin resistance in colorectal cancer [50]. Resistance to PARPi is widespread in clinical settings. More than 40% of patients with BRCA1/2 mutations do not respond to PARPi therapy, and prolonged oral administration often leads to acquired resistance. Homologous recombination deficiency (HRD), crucial for synthetic lethality, is key for cancer cell elimination. Consequently, the restoration of HRR mechanisms is a primary cause of PARPi resistance [51]. Here, we elucidated that the upregulation of SOX9 renders ovarian cancer resistant to olaparib in vitro and in vivo. To investigate the mechanism by which SOX9 regulates PARPi resistance, we conducted ChIP-seq analysis and found that SOX9 directly binds to the promoter of key DDR genes (*SMARCA4*, *UIMC1*, and *SLX4*), with enhanced bindings upon olaparib treatment. SMARCA4, also known as BRG1, is a key component of SWI/SNF chromatin-remodeling complexes and plays an important role in DDR process [52]. UIMC1 is a ubiquitin-interaction motif (UIM) containing protein that associates with a BRCA1/BARD1 complex through its interaction with CCDC98 (Abraxas). UIMC1 specifically recruits BRCA1 to DNA damage sites and works with BRCA1 in G2/M checkpoint control [53]. SLX4 contributes to genomic stability by facilitating DDR processes such as recombination, replication fork restart, telomere maintenance, and interstrand crosslink repair. Acting as an interaction scaffold or protein hub, SLX4 directly engages with essential DNA repair proteins [54, 55]. Our further research showed that USP28/SOX9 modulate the HRR activity of ovarian cancer by regulating SMARCA4, UIMC1, and SLX4 expression. Inhibition of USP28 decreased the expression of these three genes and augmented the DNA damage induced by olaparib treatment. Thus, our data indicated that USP28/SOX9 promotes olaparib resistance in ovarian cancer by regulating the DDR activity.

Experimental validation has unequivocally verified the effectiveness of targeting SOX9 as a potent therapeutic strategy in numerous malignancies, including ovarian cancer [13, 56]. Nevertheless, the lack of direct small molecule inhibitors specifically targeting SOX9 necessitates an alternative approach, focusing on the regulation of SOX9 protein stability. USP28, identified as a stabilizing enzyme for multiple oncogenic proteins, emerges as a promising candidate for small-molecule inhibition [57]. Recent investigations have demonstrated the efficacy of small-molecule deubiquitinase inhibitors in cell lines [58, 59], further reinforcing USP28 inhibition as a promising avenue for cancer treatment. In fact, various effective small molecule inhibitors specifically targeting USP28 have been developed. These inhibitors have shown strong activity in inhibiting the functions of both USP28 and its closest homolog, USP25. AZ1 is the first reported inhibitor of USP28, demonstrating an ability to bind to and inhibit USP28, while also exhibiting dual activity against USP25 [60]. AZ1 shows promising anti-tumor activity in various cancers, including liver cancer [32], lung squamous cell carcinoma (SCC) [37, 39], and ovarian cancer [61]. Vismodegib (VSM), approved for treating advanced basal cell carcinoma, inhibits the hedgehog pathway by targeting the smoothened receptor (SMO) [62]. Recent findings show that it also binds and inhibits USP28 and USP25. In colorectal cancer cells, VSM disrupts Ub-substrate binding to these proteins, inducing cytotoxic effects independently of the hedgehog pathway [63]. FT206, a patented compound from a series of substituted thienopyridine-carboxamides, is a bispecific inhibitor targeting USP28 and USP25. It shows improved drug metabolism and pharmacokinetics and exhibits potent cytotoxic effects in USP28-dependent lung SCC cells, surpassing AZ1 in efficacy. In addition to demonstrating excellent pharmacokinetic properties and potent anti-tumor effects, another advantage of FT206 is its significantly higher selectivity for USP28 over USP25 [64]. In this study, the combination of PARPi with AZ1 yielded notable improvements in PARPi outcomes. We further elaborated that the function of AZ1 depends on USP28, rather than USP25. AZ1 also augmented cell apoptosis triggered by PARPi treatment, suggesting that USP28 inhibition could offer a promising strategy for sensitizing ovarian cancer to PARPi therapy.

In conclusion, our study showed that USP28 directly interacts with SOX9 to decrease its ubiquitination and degradation, which subsequently enhances the DNA damage repair and contributes to PARPi resistance in ovarian cancer cells. Furthermore, inhibition of USP28 was shown to overcome PARPi resistance in ovarian cancer by regulating SOX9 protein stability both in vitro and in vivo. These findings contribute to a deeper understanding of PARPi resistance mechanisms and offer a potential experimental framework for addressing PARP inhibitor resistance clinically.

## Supplementary information

Supplementary information

Supplementary Table S1

original western blots

## Data Availability

The datasets used and/or analyzed during the current study are available from the corresponding author upon reasonable request.
