# Supplementary material for: USP28 promotes PARP inhibitor resistance by enhancing SOX9-mediated DNA damage repair in ovarian cancer
Source: Cell Death Dis. 2025 Apr 16;16(1):305. doi: 10.1038/s41419-025-07647-4 (PMC12003857; doi:10.1038/s41419-025-07647-4)
Supplement: Supplementary file 1 — Supplementary information [file 41419_2025_7647_MOESM1_ESM.pdf]

Supplementary Figures

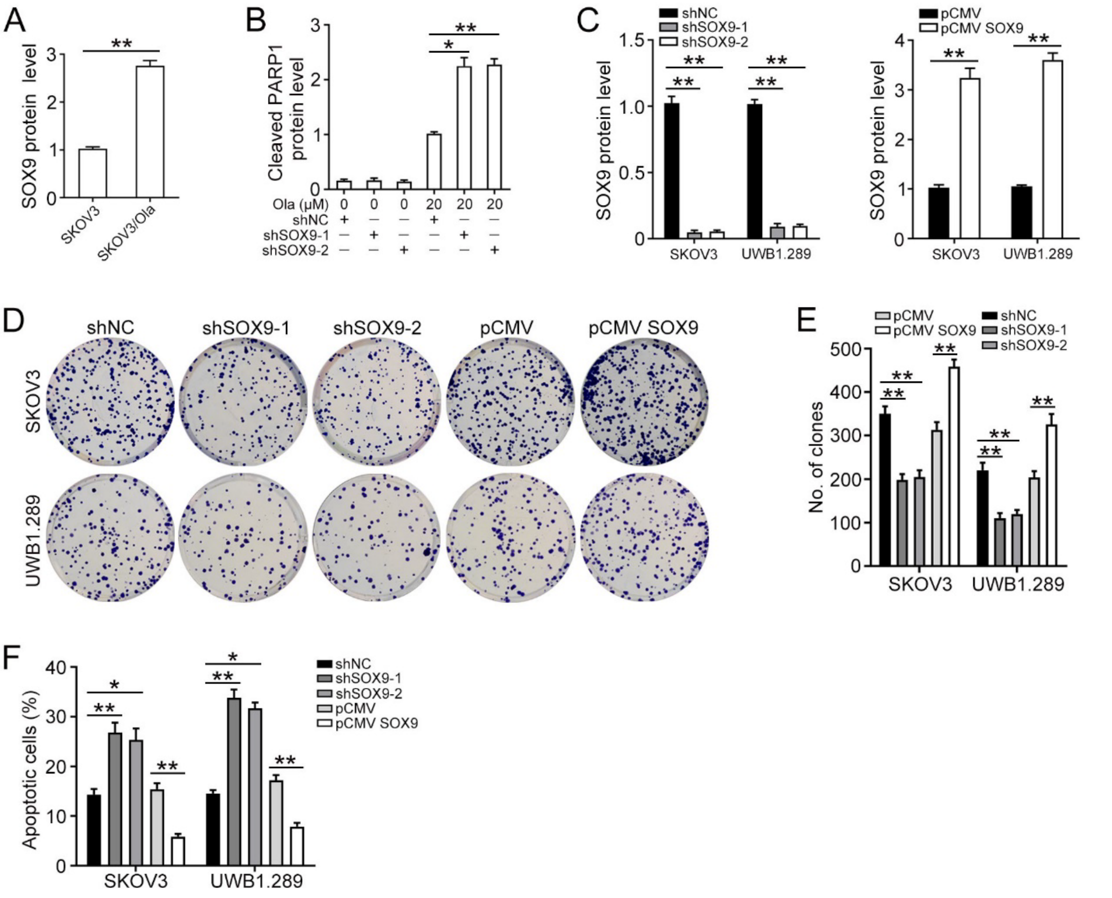

Figure S1. (A) Quantification of SOX9 protein level in Figure 1B. (B) Quantification of cleaved PARP1 protein level in Figure 1D. (C) Quantification of SOX9 protein level in Figure 1G. (D) pLKO.1 (shNC), SOX9 shRNA 1 (shSOX9-1), SOX9 shRNA 2 (shSOX9-2), pCMV, or pCMV SOX9 plasmids were stably transfected into SKOV3 and UWB1.289 cells. Clonogenic assay was used to assess the colony formation efficiency in cells treated with olaparib (SKOV3, 5 μM; UWB1.289, 1 μM). (E) Quantification of the number of clones in (D). (F) Quantification of the proportion of apoptotic cells in Figure 1I. (Data are presented as the mean ± SEM, *$p < 0.05$, **$p < 0.01$, n = 3).

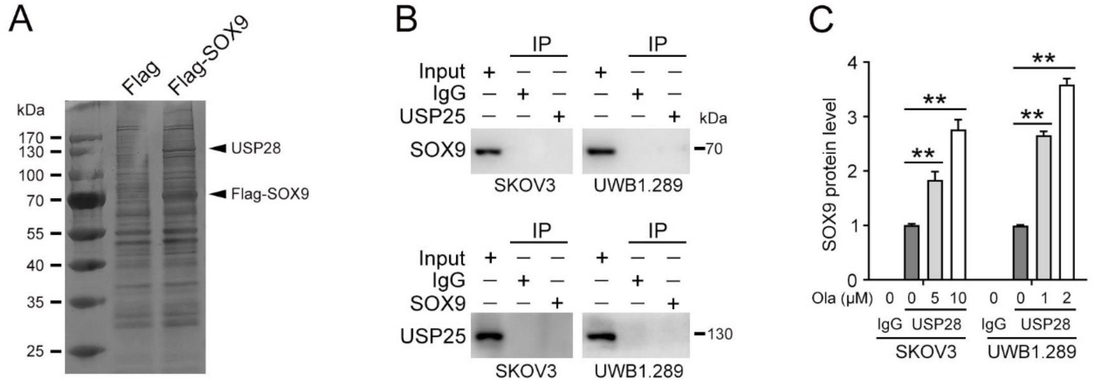

Figure S2. (A) UWB1.289 cells transfected with pCMV (Flag) or pCMV SOX9 (Flag-SOX9) were lysed and incubated with anti-Flag Beads. Then, the IPed proteins were separated on SDS-PAGE gels and silver stained. (B) Co-IP was performed to investigate the endogenous interaction between SOX9 and USP25. (C) Quantification of SOX9 protein level in Figure 2H. (Data are presented as the mean ± SEM, $**p < 0.01$, n = 3).

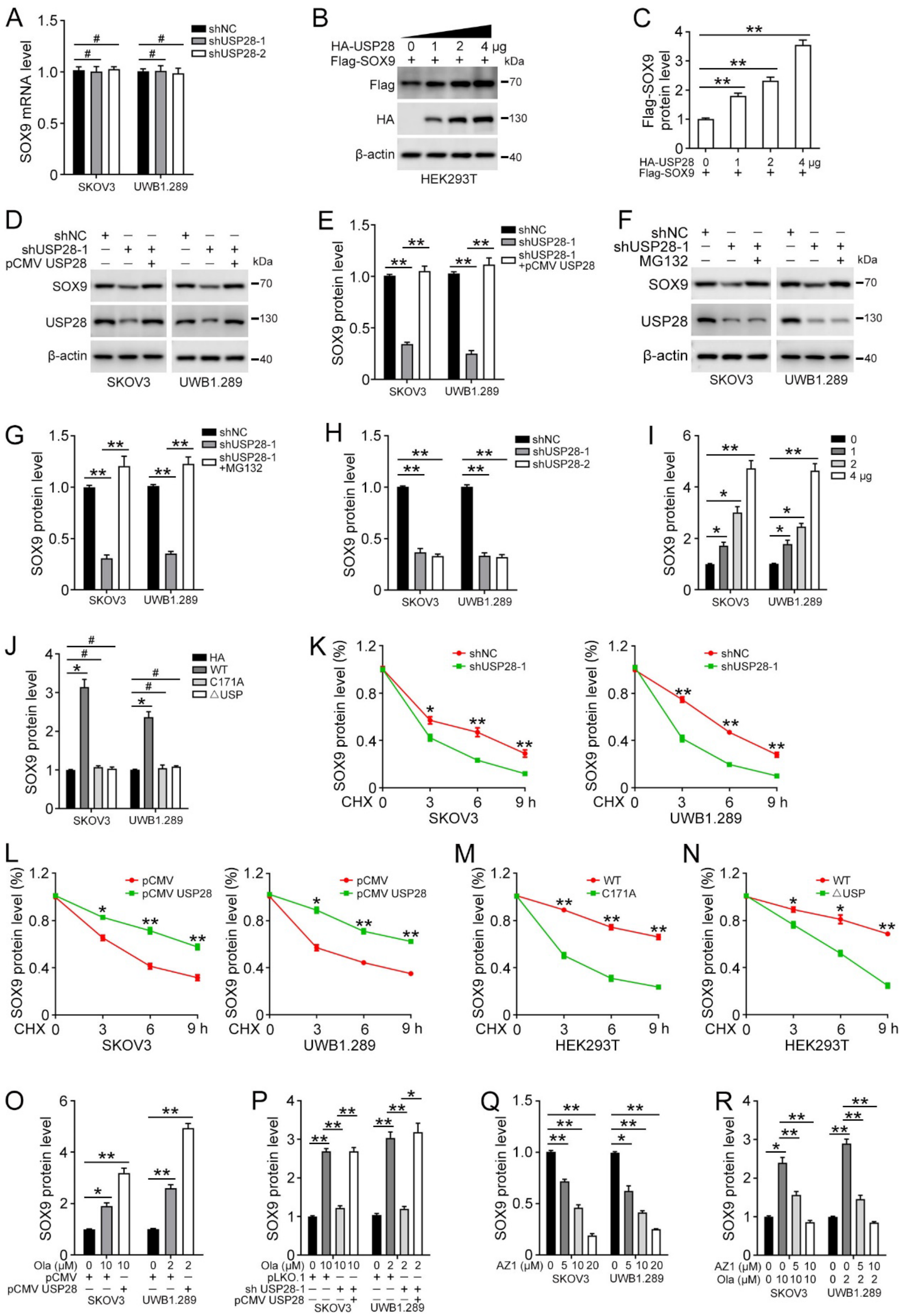

Figure S3. (A) qPCR was performed to detect the mRNA levels of SOX9 in cells with USP28 knockdown. (B) HA-USP28 (0, 1, 2, 4 μg) and Flag-SOX9 (4 μg) were co-transfected into HEK293T cells for 48 h. Western blot was performed to detect the protein levels of Flag-SOX9 and HA-USP28. (C) Quantification of Flag-SOX9

protein level in (B). (D) pLKO.1 (shNC), USP28 shRNA 1 (shUSP28-1), and pCMV USP28 were stably transfected into SKOV3 and UWB1.289 cells. Western blot was performed to detect the protein levels of SOX9 and USP28. (E) Quantification of SOX9 protein level in (D). (F) Western blot was performed to detect the protein levels of SOX9 and USP28 in cells with USP28 knockdown. MG132 (10 μM) was added 6 h before harvest. (G) Quantification of SOX9 protein level in (F). (H) Quantification of SOX9 protein level in Figure 3A. (I) Quantification of SOX9 protein level in Figure 3B. (J) Quantification of SOX9 protein level in Figure 3C. (K) Quantification of SOX9 protein level in Figure 3D. (L) Quantification of SOX9 protein level in Figure 3E. (M) Quantification of SOX9 protein level in Figure 3F. (N) Quantification of SOX9 protein level in Figure 3G. (O) Quantification of SOX9 protein level in Figure 3H. (P) Quantification of SOX9 protein level in Figure 3I. (Q) Quantification of SOX9 protein level in Figure 3J. (R) Quantification of SOX9 protein level in Figure 3K. (Data are presented as the mean ± SEM, $^{\#}p > 0.05$, $^{*}p < 0.05$, $^{**}p < 0.01$, n = 3).

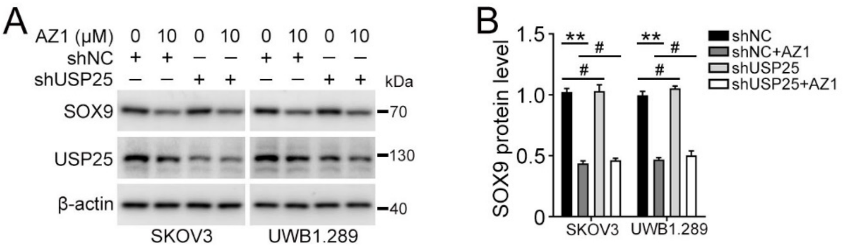

Figure S4. (A) Cells stably transfected with pLKO.1 (shNC) and USP25 shRNA (shUSP25) were treated with or without 10 μM AZ1 for 48 h. Western blot was performed to detect the protein levels of SOX9 and USP25. (B) Quantification of SOX9 protein level in (A). (Data are presented as the mean ± SEM, $^{\#}p > 0.05$, $^{**}p < 0.01$, n = 3).

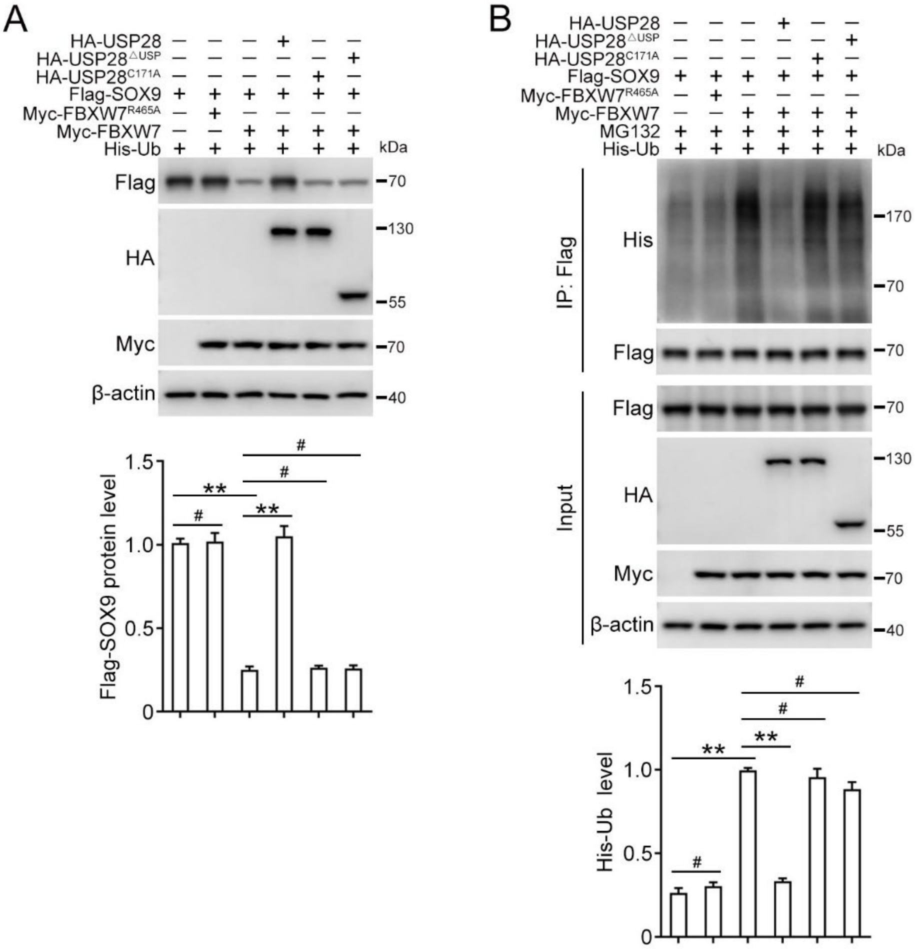

Figure S5. (A) HA-USP28, HA-USP28^{C171A}, HA-USP28$^{\Delta USP}$, Flag-SOX9, Myc-FBXW7, Myc-FBXW7^{R465A}, and His-Ub were transfected into HEK293T cells for 48 h. Western blot was performed to detect the protein levels of Flag-SOX9, HA-USP28, and Myc-FBXW7. (B) HA-USP28, HA-USP28^{C171A}, HA-USP28$^{\Delta USP}$, Flag-SOX9, Myc-FBXW7, Myc-FBXW7^{R465A}, and His-Ub were transfected into HEK293T cells for 48 h. MG132 (10 μM) was added 6 h before harvest. Co-IP was performed with anti-Flag beads and immunoblotted with the antibodies indicated. (Data are presented as the mean ± SEM, $^{\#}p > 0.05$, $^{**}p < 0.01$, n = 3).

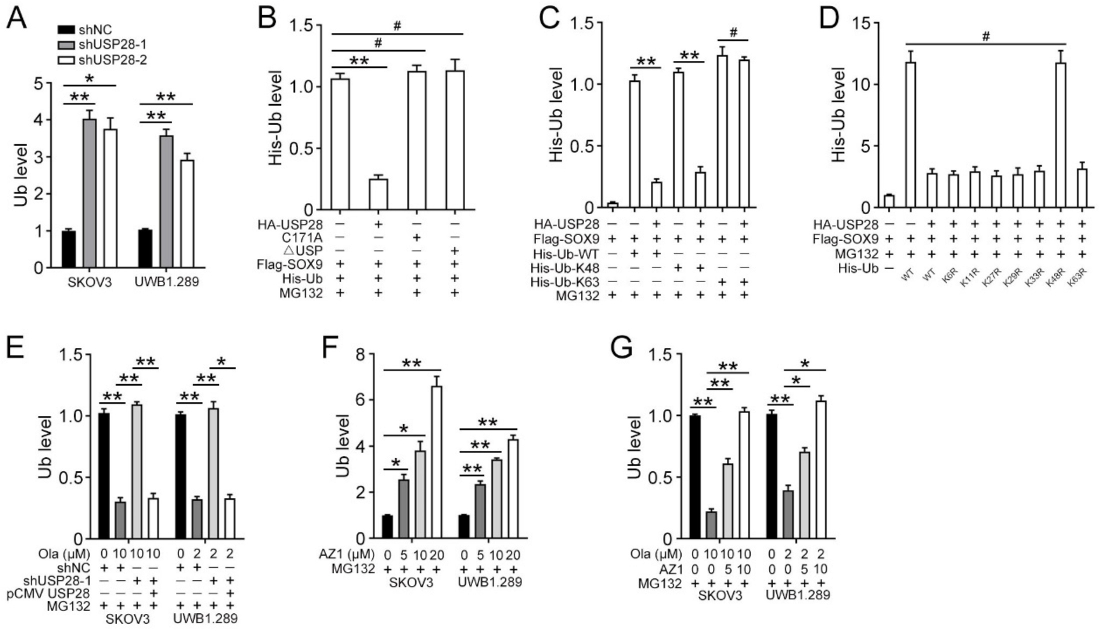

Figure S6. (A) Quantification of Ub level in Figure 4A. (B) Quantification of His-Ub level in Figure 4B. (C) Quantification of His-Ub level in Figure 4C. (D) Quantification of His-Ub level in Figure 4D. (E) Quantification of Ub level in Figure 4E. (F) Quantification of Ub level in Figure 4F. (G) Quantification of Ub level in Figure 4G. (Data are presented as the mean ± SEM, $^{\#}p > 0.05$, $^{*}p < 0.05$, $^{**}p < 0.01$, n = 3).

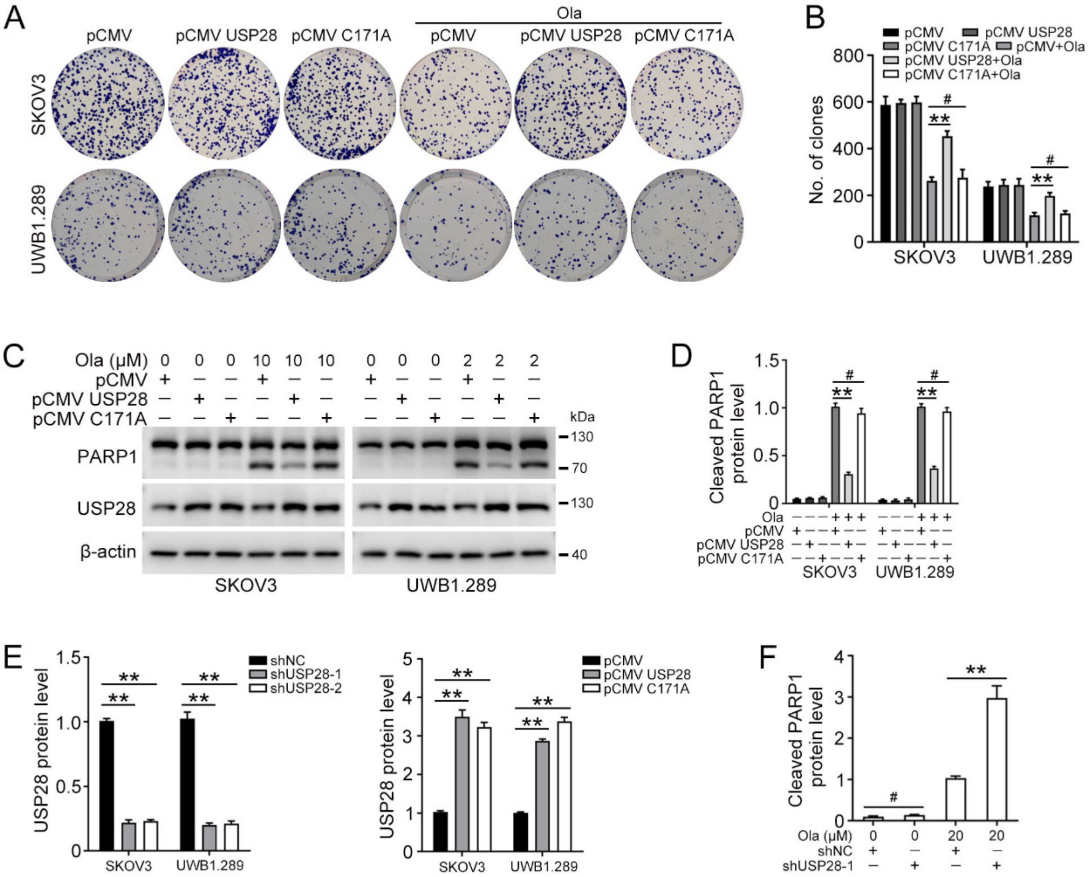

Figure S7. pCMV, pCMV USP28, or pCMV USP28^{C171A} (pCMV C171A) plasmids were stably transfected into SKOV3 and UWB1.289 cells. (A) Clonogenic assay was used to assess the colony formation efficiency of cells treated with olaparib (SKOV3, 5 μM; UWB1.289, 1 μM). (B) Quantification of the number of clones in (A). (C) Western blot was used to determine PARP1 and USP28 protein levels in cells treated with olaparib (SKOV3, 10 μM; UWB1.289, 2 μM) for 72 h. (D) Quantification of PARP1 protein level in (C). (E) Quantification of USP28 protein level in Figure 5A. (E) Quantification of cleaved PARP1 protein level in Figure 5F. (Data are presented as the mean ± SEM, $^{\#}p > 0.05$, $^{**}p < 0.01$, n = 3).

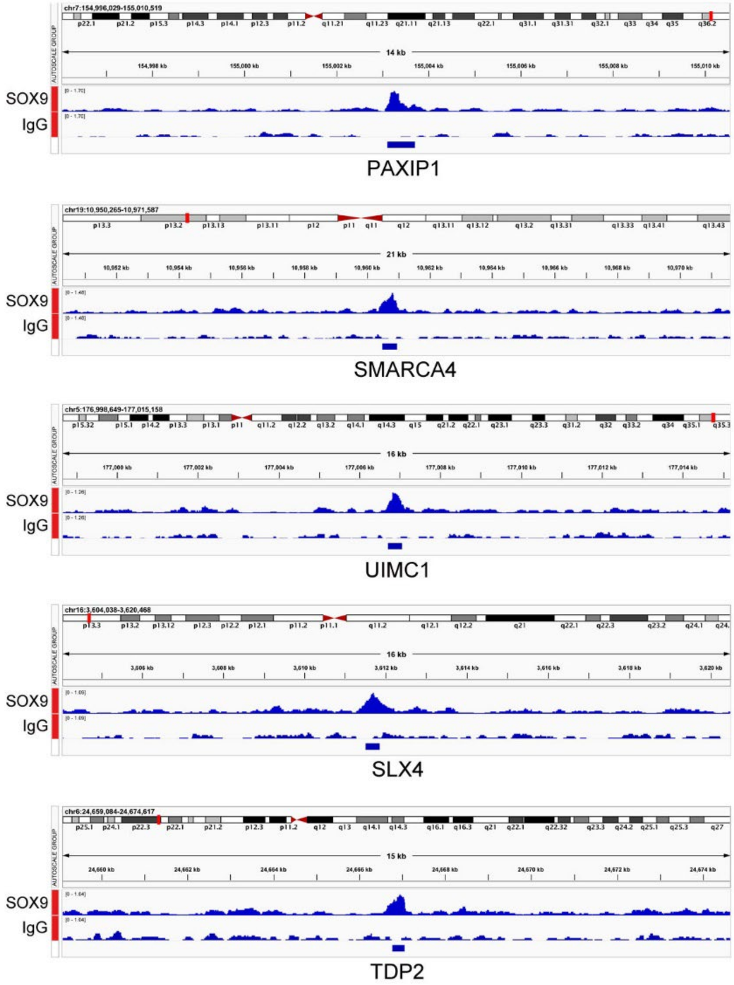

Figure S8. Analysis of SOX9 binding peaks on the promoter region based on ChIP-Seq data.

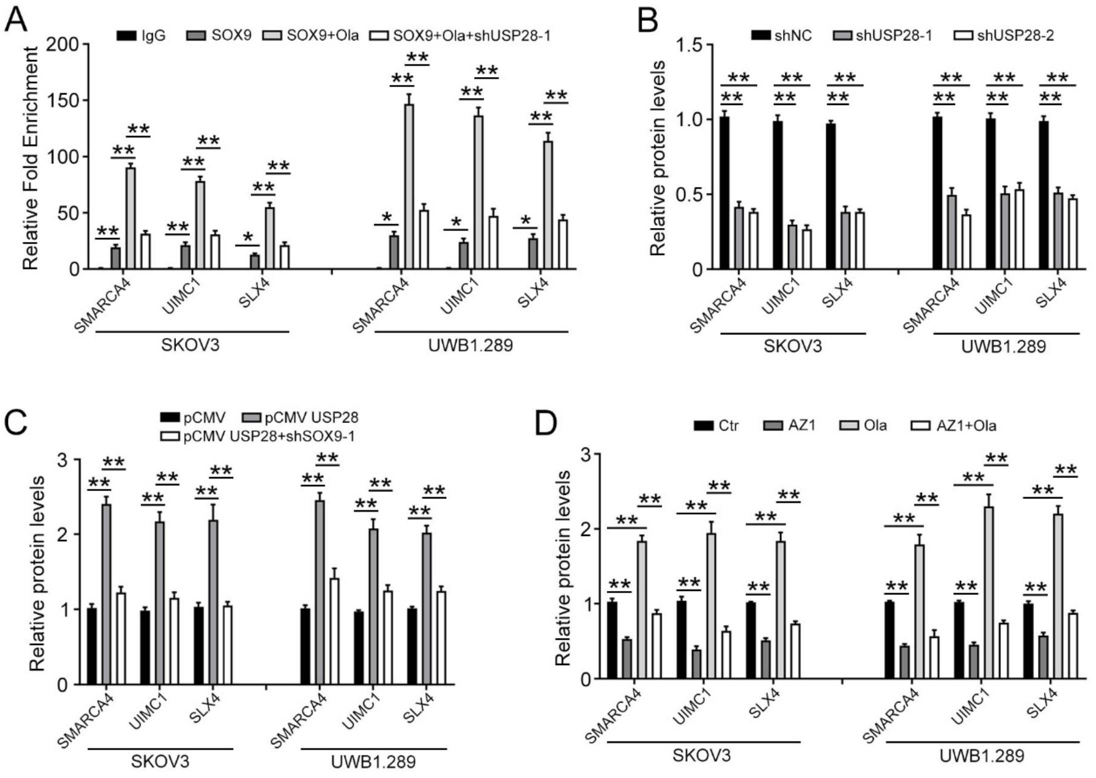

Figure S9. (A) Cells were treated with or without olaparib (SKOV3, 10 μM; UWB1.289, 2 μM) for 48 h. qPCR analysis of ChIP samples from experiments performed in SKOV3 and UWB1.289 cells using the anti-SOX9 antibody or IgG. (B) Quantification of protein levels in Figure 6G. (C) Quantification of protein levels in Figure 6H. (D) Quantification of protein levels in Figure 6I. (Data are presented as the mean ± SEM, *$p < 0.05$, **$p < 0.01$, n = 3).

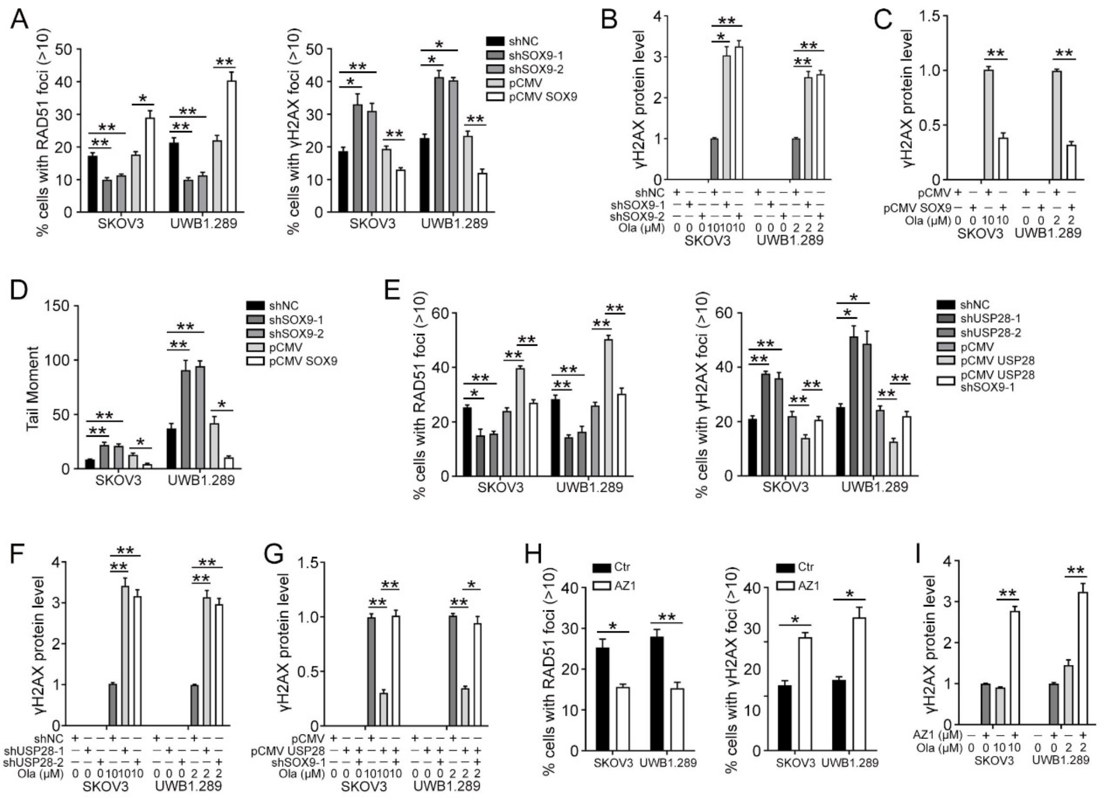

Figure S10. (A) Quantification of the percentage of cells with RAD51 foci (>10) and γH2AX foci (>10) in Figure 7A. A total of 100 cells per group were counted. (B) Quantification of γH2AX protein level in Figure 7B. (C) Quantification of γH2AX protein level in Figure 7C. (D) Quantification of the Tail Comment of comet assay in Figure 7D. A total of 100 cells per group were counted. (E) Quantification of the percentage of cells with RAD51 foci (>10) and γH2AX foci (>10) in Figure 7E. A total of 100 cells per group were counted. (F) Quantification of γH2AX protein level in Figure 7F. (G) Quantification of γH2AX protein level in Figure 7G. (H) Quantification of the percentage of cells with RAD51 foci (>10) and γH2AX foci (>10) in Figure 7H. A total of 100 cells per group were counted. (I) Quantification of γH2AX protein level in Figure 7I. (Data are presented as the mean ± SEM, **p* < 0.05, ***p* < 0.01, n = 3).

Supplementary Table S2

The primer sequences used for ChIP-qPCR.

Gene	Sequences (5' to 3')

PAXIP1-F	GGCCCGGTCCTGCGAATC
PAXIP1-R	GCCACGGAACCGCAAGGG
SMARCA4-F	CACTGCCCGGTCTTGGTCC
SMARCA4-R	TCGCCTTCTGCACGCCTC
UIMC1-F	CGGTCTCAGTTGCTTGCG
UIMC1-R	CGTGAAATTGCTGGGTCAT
SLX4-F	CCGACTCCCAGCCCCACA
SLX4-R	CGCCGCAGAGGAAGACCG
TDP2-F	CCCCAACTCCATCTTCCTGC
TDP2-R	TGACTTTCCTCGCACCCTTGT

Supplementary Table S3

The shRNA sequences targeting SOX9, USP28, and USP25

**Gene**	**Sequences (5' to 3')**
shNC	TTCTCCGAACGTGTCACGT
shUSP28-1	GCACAGAAGTTCGTTGTCATA
shUSP28-2	GACTGAAGATCATCCATTAAT
shSOX9-1	CTCCACCTTCACCTACATGAA
shSOX9-2	CTCCACCTTCACCTACATGAA
shUSP25	ACTTCTCCTGTTGACGATA

Supplementary Table S4

The primer sequences used for qRT-PCR.

**Gene**	**Sequences (5' to 3')**
PAXIP1-F	CCAGGAGGAAAGCCATGTTCAC
PAXIP1-R	CAGATGAGGACTGTGTTGCTGC
SMARCA4-F	CAAAGACAAGCACATCCTCGCC
SMARCA4-R	GCCACATAGTGCGTGTTGAGCA
UIMC1-F	GCTCTTGAGGAAAGCCATTGCTG

UIMC1-R	TTCAGTGAGCCCAGAGTCTGTG
SLX4-F	AACCCACACCTGAGTGATGTCC
SLX4-R	TCCTCTACAGCGGAGAAGCCTT
TDP2-F	GTGAAAAAGCGGCGACTTCTGTG
TDP2-R	GGCTCGAAGTAGGAGTTCAGAG
β-actin-F	CATGTACGTTGCTATCCAGGC
β-actin-R	CTCCTTAATGTCACGCACGAT
