# Supplementary material for: USP28 promotes PARP inhibitor resistance by enhancing SOX9-mediated DNA damage repair in ovarian cancer
Source: Cell Death Dis. 2025 Apr 16;16(1):305. doi: 10.1038/s41419-025-07647-4 (PMC12003857; doi:10.1038/s41419-025-07647-4)

Figure 1

1B

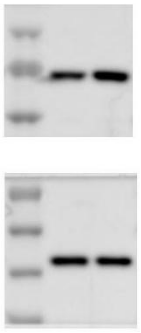

1D

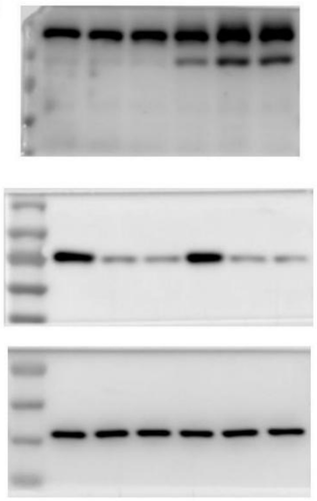

1F

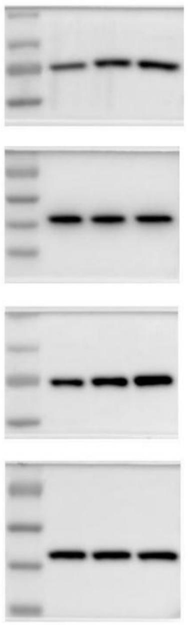

1G

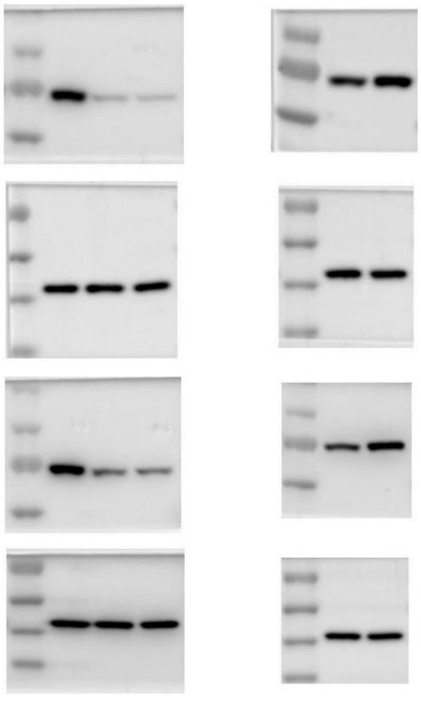

Figure 2

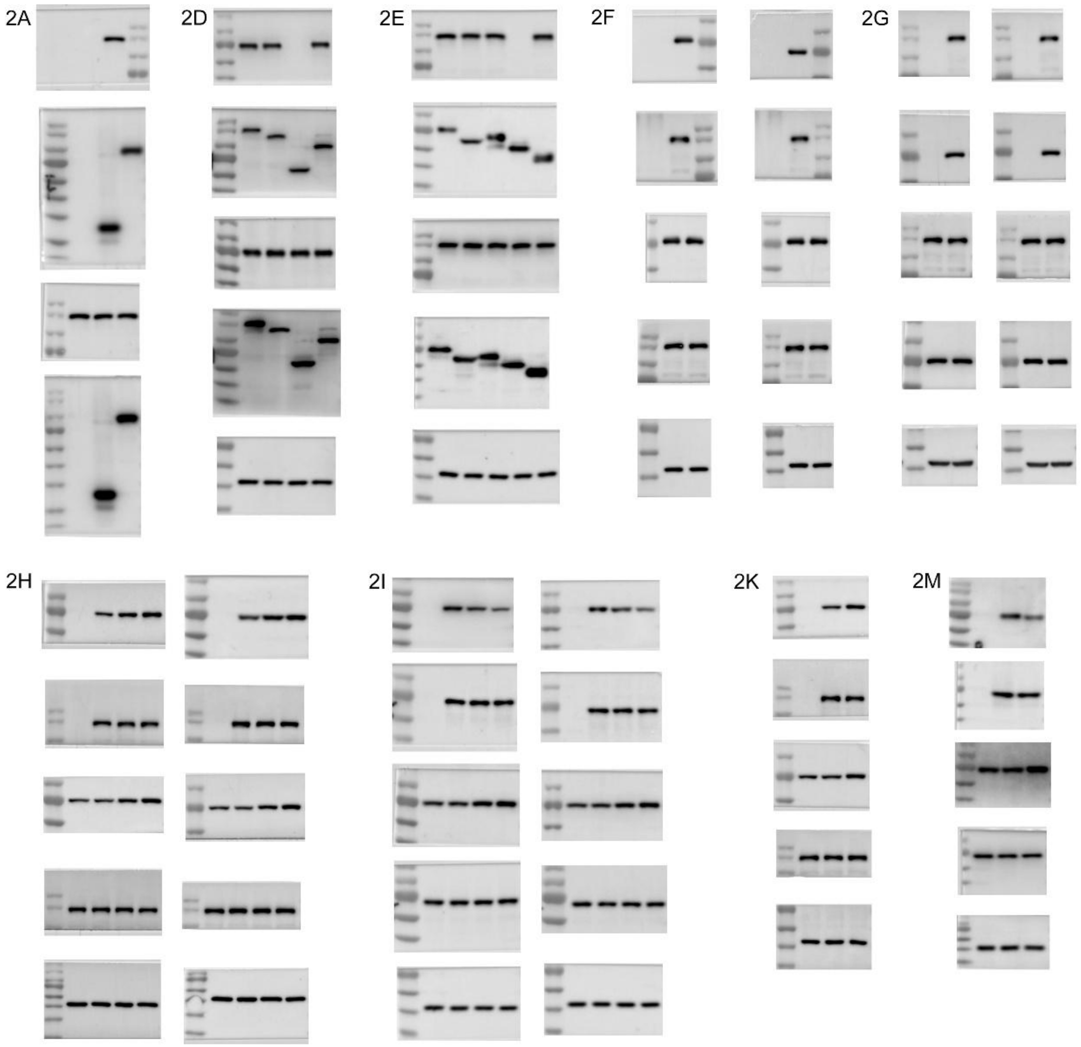

Figure 3

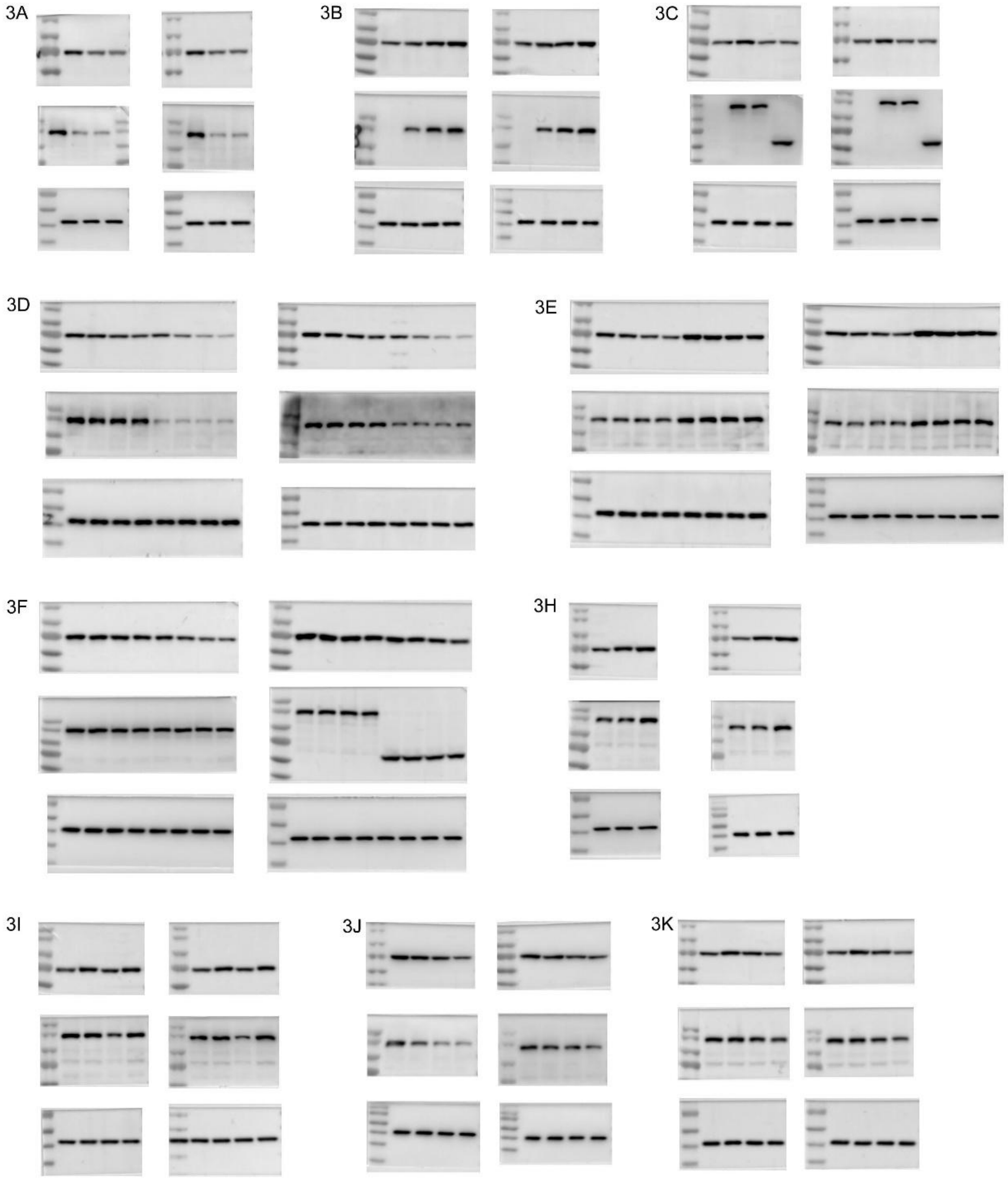

Figure 4

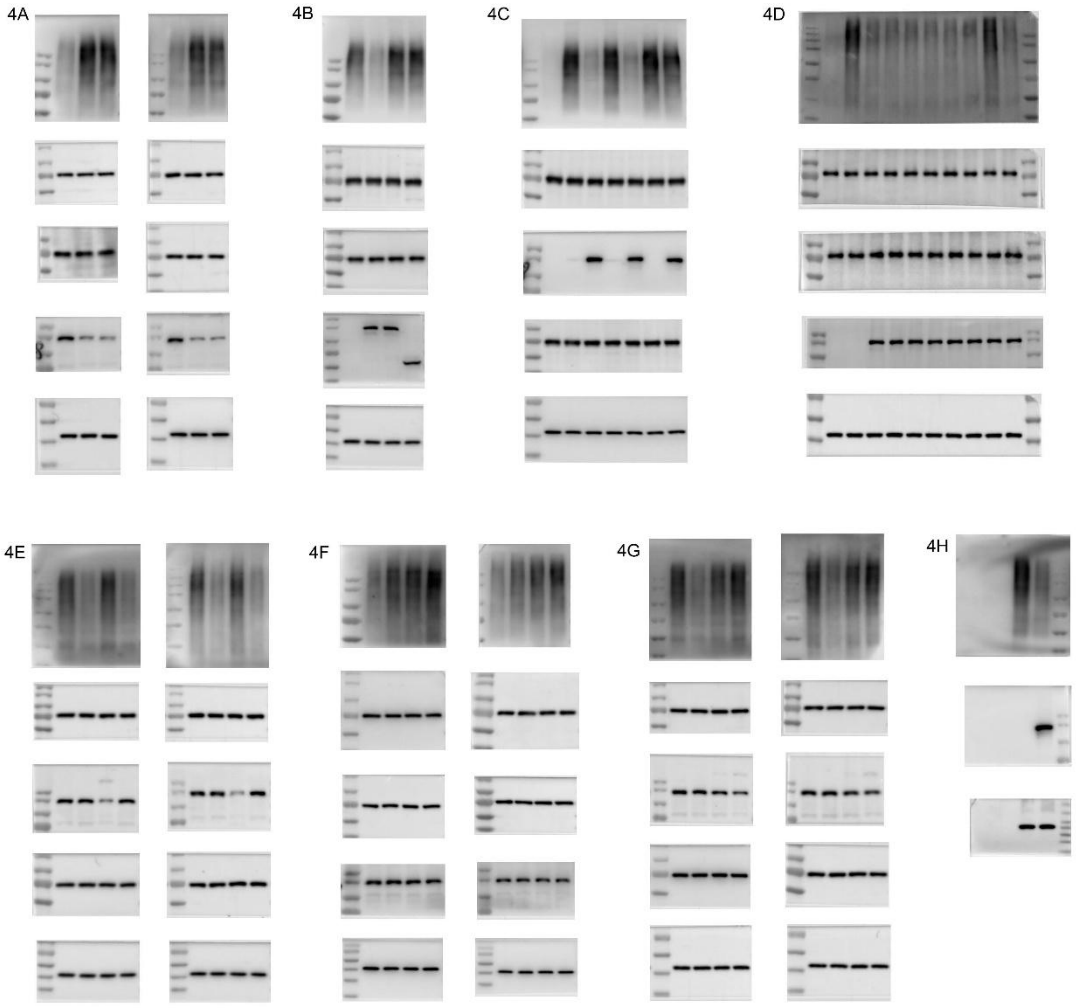

Figure 5

5A

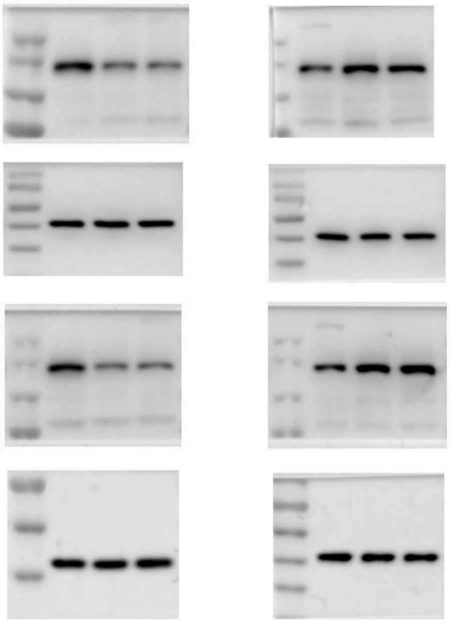

5F

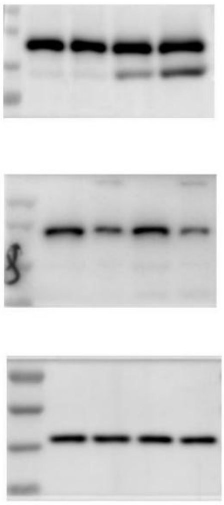

5L

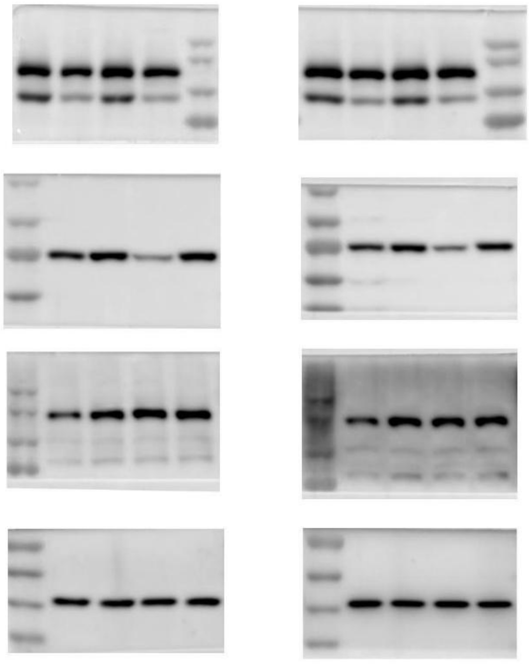

Figure 6

6E

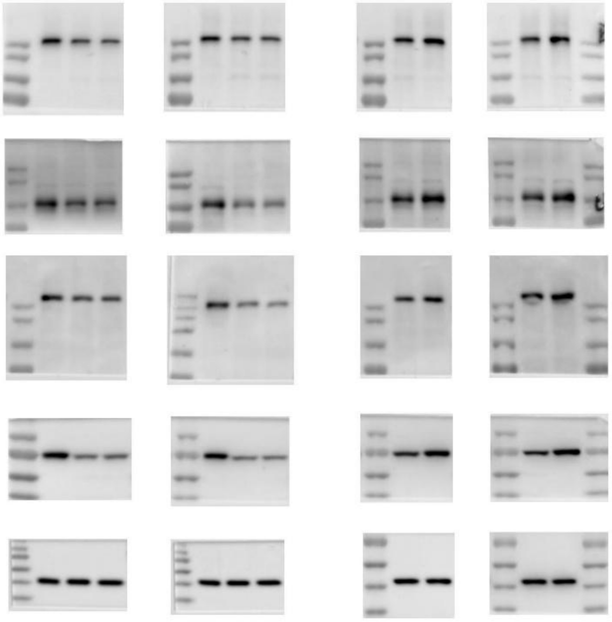

6G

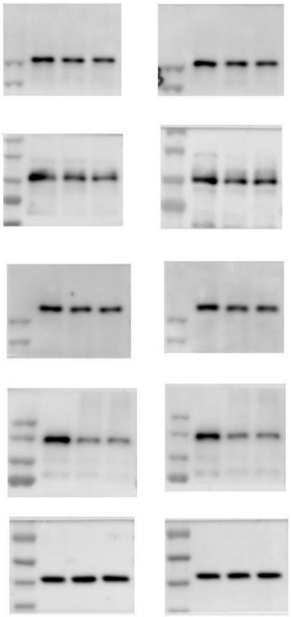

6H

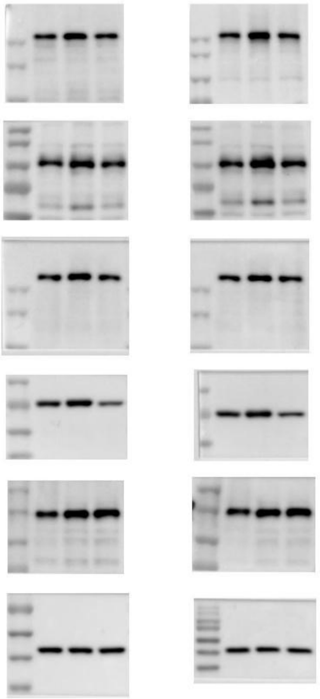

6I

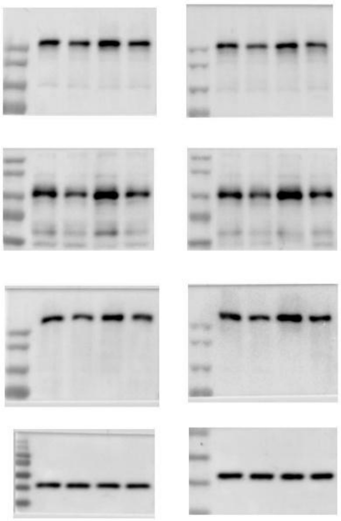

Figure 7

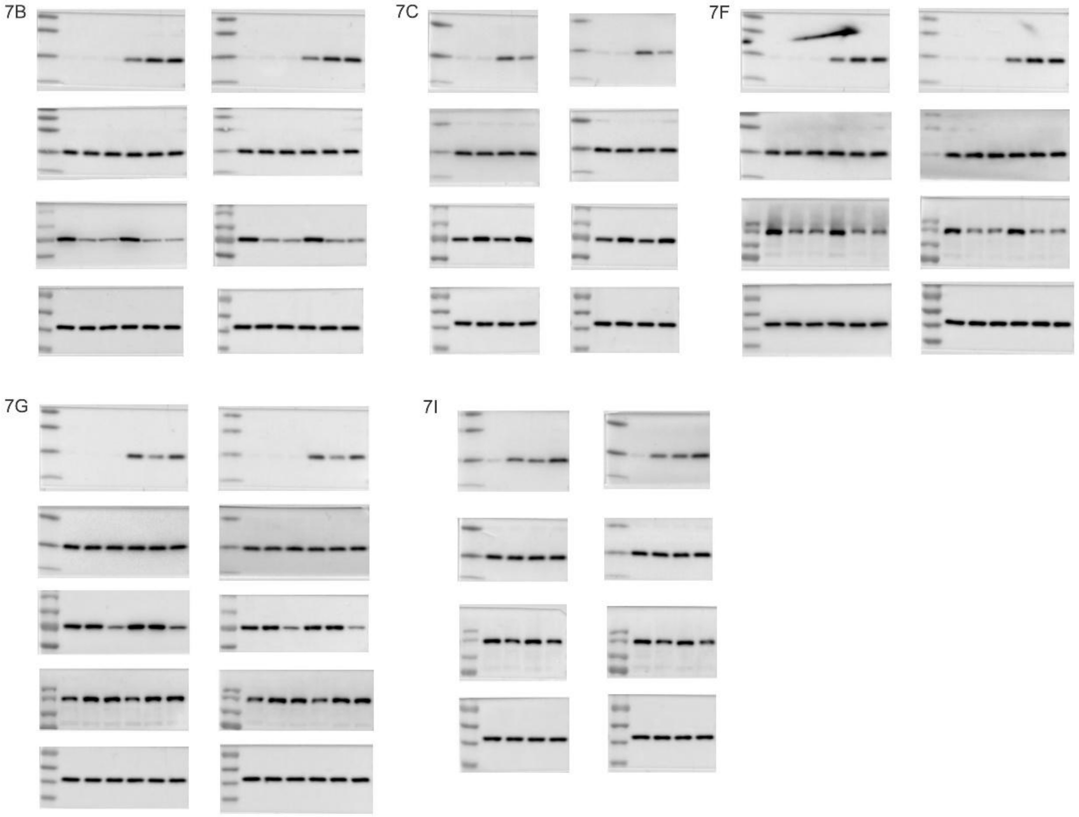

Figure 8

8F

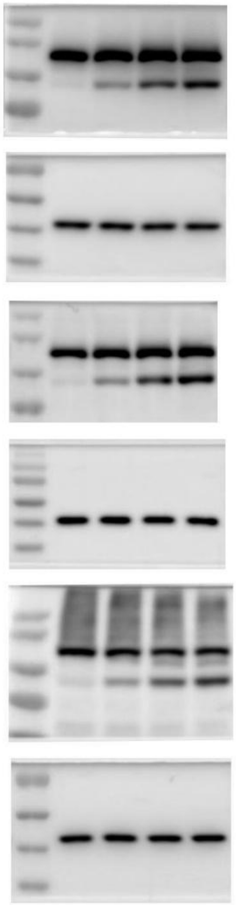

Figure S2

2B

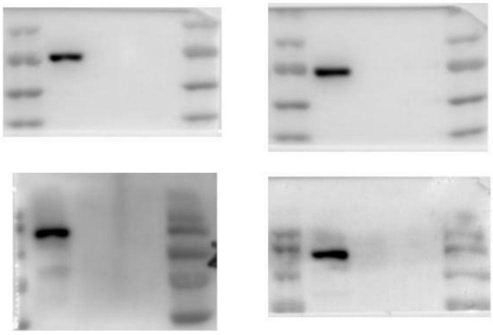

Figure S3

3B

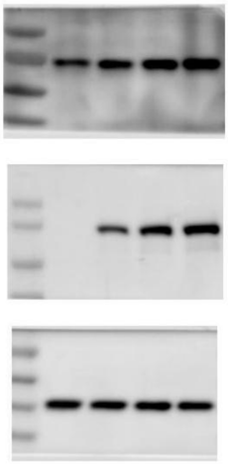

3D

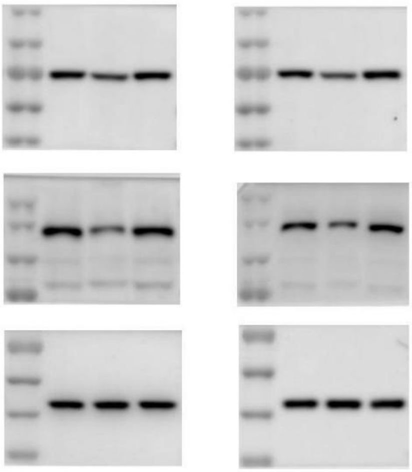

3F

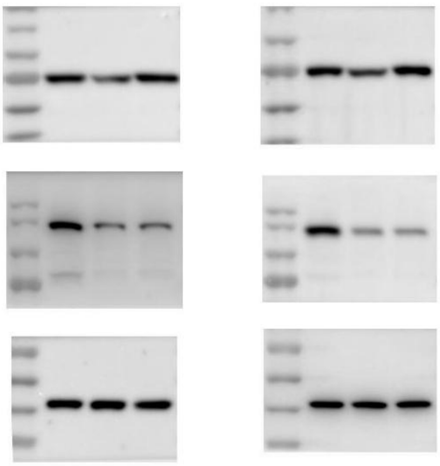

Figure S4

4A

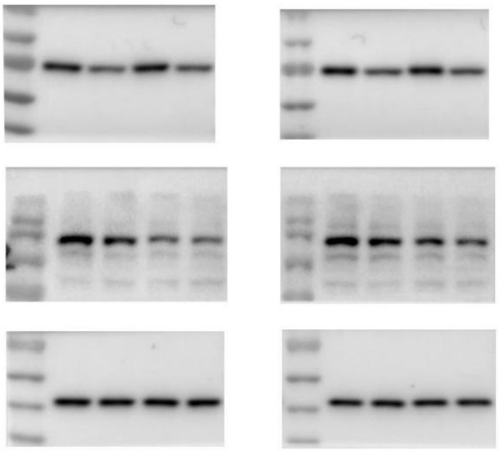

Figure S5

5A

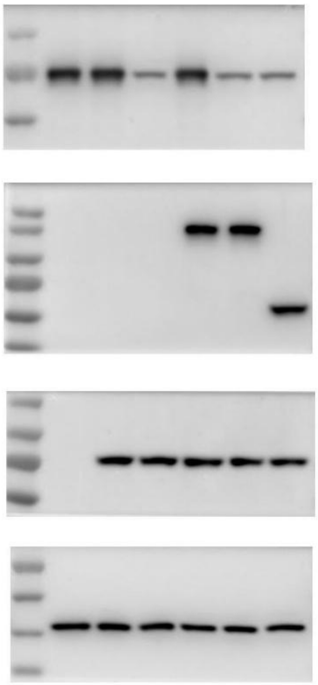

5B

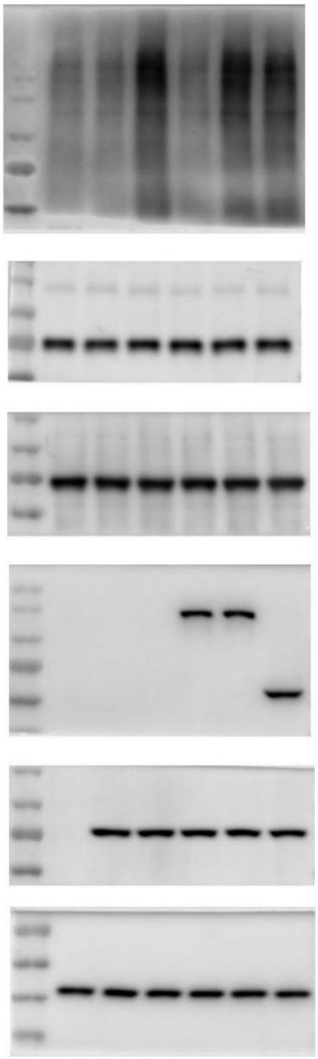

Figure S7

7C

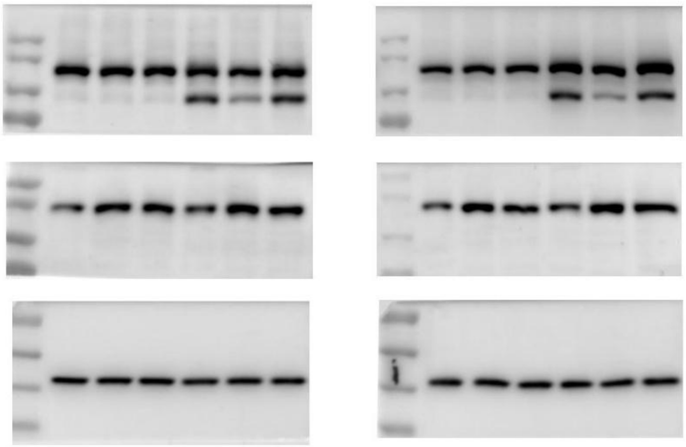

Supplement: Supplementary file 3 — original western blots [file 41419_2025_7647_MOESM3_ESM.pdf]
